# Lipid degradation and photosynthetic traits after prolonged darkness in four Antarctic benthic diatoms, including the newly described species *Planothidium wetzelii* sp. nov.

**DOI:** 10.3389/fmicb.2023.1241826

**Published:** 2023-08-31

**Authors:** Desirée P. Juchem, Katherina Schimani, Andreas Holzinger, Charlotte Permann, Nélida Abarca, Oliver Skibbe, Jonas Zimmermann, Martin Graeve, Ulf Karsten

**Affiliations:** ^1^Applied Ecology and Phycology, Institute of Biological Sciences, University of Rostock, Rostock, Germany; ^2^Botanischer Garten und Botanisches Museum Berlin, Freie Universität Berlin, Berlin, Germany; ^3^Department of Botany, Functional Plant Biology, University of Innsbruck, Innsbruck, Austria; ^4^Alfred-Wegener-Institute Helmholtz-Center for Polar and Marine Research, Ecological Chemistry, Bremerhaven, Germany

**Keywords:** Antarctica, benthic diatoms, photosynthesis, polar night, lipid consumption, plastid degradation

## Abstract

In polar regions, the microphytobenthos has important ecological functions in shallow-water habitats, such as on top of coastal sediments. This community is dominated by benthic diatoms, which contribute significantly to primary production and biogeochemical cycling while also being an important component of polar food webs. Polar diatoms are able to cope with markedly changing light conditions and prolonged periods of darkness during the polar night in Antarctica. However, the underlying mechanisms are poorly understood. In this study, five strains of Antarctic benthic diatoms were isolated in the field, and the resulting unialgal cultures were identified as four distinct species, of which one is described as a new species, *Planothidium wetzelii* sp. nov. All four species were thoroughly examined using physiological, cell biological, and biochemical methods over a fully controlled dark period of 3 months. The results showed that the utilization of storage lipids is one of the key mechanisms in Antarctic benthic diatoms to survive the polar night, although different fatty acids were involved in the investigated taxa. In all tested species, the storage lipid content declined significantly, along with an ultrastructurally observable degradation of the chloroplasts. Surprisingly, photosynthetic performance did not change significantly despite chloroplasts decreasing in thylakoid membranes and an increased number of plastoglobules. Thus, a combination of biochemical and cell biological mechanisms allows Antarctic benthic diatoms to survive the polar night.

## 1. Introduction

The polar regions represent unique ecosystems characterized by low temperatures, ice and snow cover, and pronounced seasonal fluctuations in light availability with long periods of complete darkness during the polar night. The abiotic factor of light is especially challenging for autotrophic primary producers living in polar regions. Regional ice and snow cover can further extend the dark period for organisms (Cottier and Potter, 2020). If sea ice is covered with snow, photon fluence rates can be reduced to 2% of the solar surface radiation, leaving the organisms underneath exposed to extremely low light conditions or even darkness for up to 10 months in certain regions (Karsten et al., 2019a). Polar algae must, therefore, tolerate both very high light conditions after ice break-up and extremely low irradiances (Gómez et al., 2009; Zacher et al., 2009) and must have high adaptability to such fluctuating conditions in combination with always enhanced photosynthetic efficiency and plasticity (Longhi et al., 2003).

Diatoms are the most species-rich group of microalgae and dominate well-mixed water columns across all oceans as well as benthic algal communities of shallow-water soft bottoms, and rocky substrates (Lacour et al., 2019). They are responsible for ~20–25% of the global and 40–45% of the marine primary production (Nelson et al., 1995; Field et al., 1998), which reflects their important role in marine food webs and in the global carbon cycle as well as other biochemical cycles (Field et al., 1998; Armbrust, 2009). In depths down to 30 m, the primary production of microphytobenthic communities forms the main food source for benthic suspension or deposit feeders and thus plays a major role in the ecology of polar coastal habitats (Glud et al., 2009). Carbon budget measurements in Young Sound, Greenland, showed almost 50% of primary production originating from microphytobenthos (Glud et al., 2002), which could also be confirmed for the Arctic Kongsfjorden (Svalbard, Norway; Woelfel et al., 2010). In Antarctica, McMinn et al. (2012) described that in <5 m of water depth, benthic primary production exceeds that of phytoplankton or sea ice algae. Consequently, benthic diatoms play a crucial role in polar ecosystems and coastal food chains (Glud et al., 2009) but are still less studied compared to their planktonic or ice-associated counterparts. Marine Antarctic benthic diatoms are still poorly studied in terms of biodiversity, biogeography, and ecology, while for freshwater habitats, comprehensive datasets exist (Verleyen et al., 2021; Al-Handal et al., 2022).

Diatom taxa from different Arctic and Antarctic habitats, such as sea ice, water column, and soft bottom, are reported to survive long periods of complete darkness (Schaub et al., 2017, and literature therein). The species-specific maximum survival periods are highly variable, ranging from 3 months to 1 year, and benthic diatoms have been reported with the longest survival times (Antia, 1976). Experiments on the dark survival potential of different Arctic benthic diatom species indicated a high tolerance, i.e., survival for up to 5 months without light. Although chloroplast volume was strongly reduced with increasing dark treatment, *Cylindrotheca closterium* (Ehrenberg) Reimann and J. C. Lewin and *Surirella* cf. *minuta* showed high growth rates after a few days of lag phase after re-irradiation (Karsten et al., 2012; Schlie and Karsten, 2017). During the polar night, the reallocation of energy toward maintenance metabolism through the decomposition of organelle components or lipid droplets seems to be a key process for survival in benthic polar diatoms. The lag phase after transfer to light can be interpreted as a recovery period, in which diatom cells rebuild cellular structures and metabolic activity (Karsten et al., 2012). In order to maintain viability, not only organelles but also membranes and DNA must remain intact (McMinn and Martin, 2013). Benthic diatoms with intact plasmalemma can be distinguished from those with permeabilized membranes using the nucleic acid stain SYTOX Green, which only passes through compromised or damaged membranes, and stains the nucleus, leading to enhanced fluorescence under blue light excitation (Karsten et al., 2019a,b, and references therein). Application of the SYTOX Green stain to dark-incubated Arctic *Navicula directa* (W. Smith) Brébisson indicated that >95% of all cells exhibited intact membranes, even after 5 months of darkness, and hence, the high degree of membrane integrity contributed to long-term dark tolerance (Karsten et al., 2019a,b). Lower temperatures generally reduce the metabolic activity of all organisms, thereby enhancing the dark survival potential of polar benthic diatoms. Reeves et al. (2011) reported for Antarctic sea ice diatoms a reduced dark survival time at 10°C compared to −2°C but no negative effect at 4°C. *Fragilariopsis cylindrus* (Grunow ex Cleve) Helmcke and Krieger survived 60 days of darkness at both −2 and 4°C but only 7 days at 10°C (Reeves et al., 2011).

The physiological state in which polar diatoms survive the darkness and the underlying metabolic processes is still almost unstudied. In the few diatoms studied, different mechanisms have been described for coping with the polar night (McMinn and Martin, 2013). Those include the reduction of metabolic activity (Palmisano and Sullivan, 1982), the utilization of stored energy products (Palmisano and Sullivan, 1982; Schaub et al., 2017), formation of resting stages (Durbin, 1978; McQuoid and Hobson, 1996), and a mixotrophic lifestyle (Hellebust and Lewin, 1977; Tuchman et al., 2006). These adaptive mechanisms are not considered to be mutually exclusive, as they probably vary in relative importance among polar algae (Palmisano and Sullivan, 1983). The utilization of energy storage products, such as the typical reserve carbohydrate chrysolaminarin or lipids (triacylglycerol), the latter being stored in cell vacuoles and/or in cytoplasmic lipid droplets, could sustain the cellular maintenance metabolism during long periods of darkness. Schaub et al. (2017) confirmed in the Arctic benthic diatom *Navicula* cf. *perminuta* the preferential utilization of the stored lipid compound triacylglycerol during prolonged dark periods, but also the pool of free fatty acids.

Diatoms are well-known for their metabolic strategy to store energy as lipids, often as neutral triacylglycerol, which consist of a glycerin backbone esterified with three fatty acids and which are deposited in densely packed lipid droplets intracellularly located in the cytoplasm (Hu et al., 2008; Leyland et al., 2020). Lipids can store more energy per molecule compared to carbohydrates or proteins (Morales et al., 2021), and hence, high proportions of such lipid bodies have been described in polar diatoms from the water column and sea ice, particularly in late autumn prior to the onset of the polar night (Fryxell, 1989; Fahl and Kattner, 1993; Zhang et al., 1998), while Antarctic benthic diatoms are almost unstudied.

For the present study, five benthic diatom strains from King George Island, Antarctic Peninsula, were morphologically and molecularly identified and examined using physiological, cell biological, and biochemical methods to better understand the underlying mechanisms for coping with the polar night. Over a period of 3 months of experimental dark treatment, we evaluated photosynthesis, respiration, and chlorophyll *a* content, as well as ultrastructure, lipid body size, and volume in combination with fatty acid concentrations prior to and after the dark treatment. Based on previous studies in Arctic benthic diatoms (Schaub et al., 2017, and references therein), we expected a strong involvement of storage lipids in the survival of the polar night in Antarctic benthic diatoms. In addition, our study provides transmission electron microscopic images of these ecologically important primary producers to our knowledge for the first time.

## 2. Materials and methods

### 2.1. Description of the study site with ecological characterization

**Figure 1:**
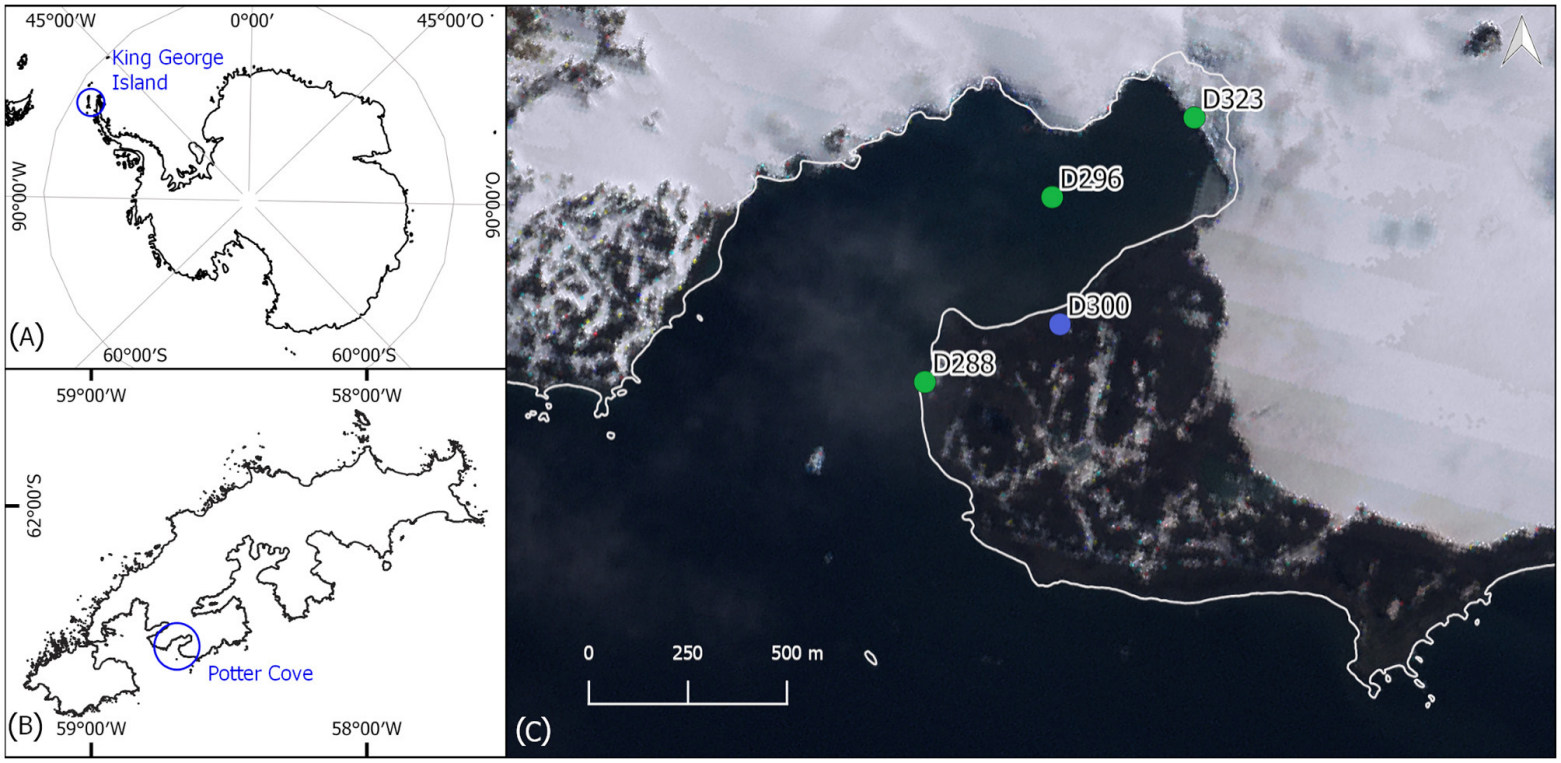
**(A)** Map of Antarctica; **(B)** map of King George Island; **(C)** map of Potter Cove, with the four sampling locations. The blue point represents the freshwater sampling site and green points represent marine sampling locations. Base map: Landsat Image Mosaic of Antarctica (LIMA).

Sediment surface samples were taken from 28 January to 15 February 2020 from four study sites near the Argentinian research station Carlini Base (S 62°14′17.45”, W 58°40′2.19”) at Potter Cove on King George Island, Antarctic Peninsula, and used for benthic diatom isolation (Figure 1, Table 1). The marine culture D288_003 originated from sample location D288 (62° 14′ 30.55” S, 58° 40′ 54.96” W) and was isolated from a biofilm of an intertidal rock pool. D296_001 was isolated from a marine sample at the inner part of Potter Cove (62° 13′ 43.61“ S, 58° 39′ 49.36” W), at 15 m depth, from an epipsammic community. The marine culture D323_018 originated from a sample location closer to the glacier front in the inner part of the bay at 10 m water depth from an epipsammic community (62°13′25.68” S, 58°38′33.50” W). Both limnic isolates (D300_015 and D300_025) were established from biofilms on top of stones in a freshwater drinking reservoir (62° 14′ 16.30” S, 58° 39′ 44.10” W).

**Table 1:** List of benthic diatom strains established from Antarctic marine and freshwater samples with scientific names, information on dimensions of the valves, striae density, sequenced marker genes, and accession numbers, RV, raphe valve; SV, sternum valve.

**Strain**	**Scientific name**	**Water type**	**Length/ diameter (μm)**	**Width/ pervalvar axis (μm)**	**Striae in 10 μm**	**Marker genes**	**Accession number *rbc*L**	**Accession number 18SV4/18S**
D288_003	*Navicula criophiliforma*	Marine	24.2–52.4	5.8–8.5	11–12	18S V4, *rbc*L	OX258986	OX259166
D296_001	*Chamaepinnularia gerlachei*	Marine	17.1–20.6	4.1–5.4	18–20	18S, *rbc*L	OX258987	OX258985
D323_018	*Melosira* sp.	Marine	18.6–20.9	19.4–22.0	-	18S V4, *rbc*L	OR036645	OR042180
D300_015	*Planothidium wetzelii* sp. nov.	Freshwater	10.9–11.3	5.6–6.1	16–18 (RV)	18S V4, *rbc*L	OX258989	OX259168
					17–18 (SV)			
D300_025	*Planothidium wetzelii* sp. nov.	Freshwater	17.8–18.8	6.0–6.3	14–15 (RV)	18S V4, *rbc*L	OR036646	OR042181
					15–16 (SV)			
D300_019^*^	*Planothidium wetzelii* sp. nov.	Freshwater	11.1–11.8	5.8–6.7	16–18 (RV)	18S V4, *rbc*L	OR036648	OR042183
					16–17 (SV)			
D300_020^*^	*Planothidium wetzelii* sp. nov.	Freshwater	17.5–18.5	5.8–6.5	14–15 (RV)	18S V4, *rbc*L	OR036647	OR042182
					14–15 (SV)			

^*^Strains were only used for the description of the new species.

At Carlini Base, various abiotic parameters were recorded. The minimum water temperature was −1.69°C in the inner part of Potter Cove and −1.4°C in the outer part, while the maximum temperature at both sites was 2.89 and 1.98°C, respectively. Furthermore, the salinity of the outer cove was stable at ~33.5 S_A_, while the salinity in the inner cove can decline to 29.6 S_A_ because of meltwater run-off (Hernández et al., 2019). Light conditions are diurnally and seasonally highly variable, and more recent data were published by Hoffmann et al. (2019).

### 2.2. Culture establishment

The diatom cells were isolated from aliquots of environmental samples to establish clonal cultures. Under an inverse light microscope (Olympus, Hamburg, Germany), single cells were transferred using microcapillary glass pipettes onto microwell plates containing culture medium [Guillard's f/2 medium (Guillard and Ryther, 1962) or Walne's medium (Walne, 1970), 34 S_A_ for marine samples and 1 S_A_ for freshwater samples]. All samples and isolated diatom cells from Antarctica were maintained at 5–7°C at the Botanische Museum Berlin. Irradiation was accomplished by white LEDs with 5,000 K under a 16/8 day/night cycle with several dark phases during the day to prevent photo-oxidative stress. After the successful establishment of clonal cultures, they were separated into subsamples for DNA extraction, morphological analysis, and ecophysiological, biochemical, and cell biological experiments. For all experimental approaches, diatom cultures were transferred to the University of Rostock and cultured in sterile-filtered Baltic Sea water, enriched with Guillard's f/2 medium (Guillard and Ryther, 1962) and metasilicate (Na_2_SiO_3_ • 5 H_2_O; 10 g 100 mL^−1^) to a final concentration of 0.6 mM (further referred to as culture media). The salinity of 33 S_A_ for the marine cultures was adjusted by adding artificial sea salt (hw-Marinemix^®^ professional, Wiegandt GmbH, Germany), while 1 S_A_ for the limnic cultures was achieved by dilution with deionized water. The media were regularly changed to replenish nutrients.

All stock cultures at the University of Rostock were kept at 8–9°C and 15–20 μmol photons m^−2^ s^−1^ under a 16/8-h light/dark cycle [Osram Daylight Lumilux Cool White lamps L36W/840 (Osram, Munich, Germany)].

### 2.3. Taxonomic identification

In order to obtain clean diatom frustules for species identification, material harvested from the clonal cultures was treated with 35% (v/v) hydrogen peroxide at room temperature to oxidize the organic material, followed by several washing steps with distilled water. To prepare permanent slides for light microscopy (LM) analyses, the cleaned material (frustules and valves) was dispersed on cover glasses, dried at room temperature, and embedded with the high refraction index mounting medium Naphrax^®^.

For each culture, 15–20 cells were measured for morphometry. Observations were conducted with a Zeiss Axioplan Microscope equipped with differential interference contrast (DIC) using a Zeiss 100x/1.30 Plan Apochromat objective, while microphotographs were taken with an AXIOAM MRc camera. Aliquots of cleaned sample material for scanning electron microscopy (SEM) observations were mounted on stubs and observed under a Hitachi FE 8010 scanning electron microscope operated at 1.0 kV.

#### 2.3.1. DNA extraction, amplification, sequencing, and processing

Clonal diatom material from log phase cultures was transferred to 1.5 mL tubes. DNA was isolated using the NucleoSpin^®^ Plant II Mini Kit (Macherey and Nagel, Düren, Germany), following the manufacturer's instructions. DNA fragment size and concentrations were evaluated via gel electrophoresis (1.5% agarose gel) and NanoDrop^®^ (Peqlab Biotechnology LLC; Erlangen, Germany), respectively. DNA samples were stored at −20°C until further use and finally deposited in the Berlin collection of the DNA Bank Network (Gemeinholzer et al., 2011).

DNA amplification was conducted by polymerase chain reaction (PCR) after Zimmermann et al. (2011) for the V4 region of 18S. For strain D296_001, the whole 18S gene was amplified after Jahn et al. (2017). The protein-coding plastid gene *rbc*L was amplified by Abarca et al. (2014). PCR products were visualized on a 1.5% agarose gel and cleaned with MSB Spin PCRapace^®^ (Invitek Molecular GmbH; Berlin, Germany) following the manufacturer's instructions. DNA content was measured using NanoDrop^®^ (Peqlab Biotechnology). The samples were normalized to a total DNA content >100 ng μL^−1^ for sequencing. Sanger sequencing of the PCR products was conducted bidirectionally by StarSEQ^®^ (GENterprise LLC; Mainz, Germany).

Electropherograms obtained by Sanger sequencing were checked manually. The resulting reads from both sequencing directions overlapped, and the fragments were assembled in PhyDE^®^ (Müller et al., 2010) to obtain the final sequences of the amplified markers.

The genetic differences of the newly described *Planothidium* to other species of this genus were investigated based on the 18S V4 and *rbc*L sequence matrices using MEGA 11 (Tamura et al., 2021) and the implemented p-distance option. Therefore, our dataset was complemented with 21 sequences of *Planothidium* from NCBI (Supplementary Tables 1, 2) and that of *P. tujii* C. E. Wetzel and Ector (molecular data supplied by A. Tuji). The alignments were trimmed to 437 bp for 18S V4 and 988 bp for *rbc*L.

#### 2.3.2. Data curation

Vouchers and DNA of all strains were deposited in the collections at Botanischer Garten und Botanisches Museum Berlin, Freie Universität Berlin (B). DNA samples were stored in the Berlin DNA bank and were available via the Genome Biodiversity Network (GGBN; Droege et al., 2016). All sequences were submitted to GenBank. All cultures were available from the authors at the culture collection of the Department of Applied Ecology and Phycology, University of Rostock.

### 2.4. Experimental part

After cultivation for 4–6 weeks to achieve sufficient biomass for the experiments, each strain was divided into different Erlenmeyer flasks. From the control condition (T0, control), three replicate samples were harvested for evaluation, and an additional three replicates were transferred to 3 months of darkness (T3) at 5°C. Cells from both T0 and T3 treatments were investigated concerning physiological, biochemical, and cell biological traits.

#### 2.4.1. Photosynthetic efficiency

The efficiency of energy transfer in the diatom chloroplasts from the antenna to photosystem II (PS II) allows conclusions about the cell viability and the physiological state of the cells. It was measured using a pulse amplitude modulation (PAM) fluorimeter (PAM-2500, Heinz Walz GmbH, Effeltrich, Germany). The maximum quantum yield [Y(II)_max_] of PS II was calculated by detecting the ground fluorescence (F_0_) of the dark-adapted samples and the maximal fluorescence (F_m_) after an oversaturating light pulse:


$$\begin{array}{l} {Y\left(II\right)}_{\max}=\frac{F_{v}}{F_{m}}=\frac{\left(F_{m}-F_{0}\right)}{F_{m}} \end{array}$$

For measurements, a cooling block was used and set to the dark incubation temperature of 5°C to avoid temperature stress in the Antarctic benthic diatom samples. On the cooling block, 25-mm glass fiber filters (GF/6, Whatman, Little Chalfont, UK) were wetted with the respective culture medium. In a dark working space, a uniformly dense diatom cell layer was dripped on the filter and immediately measured. For detecting F_0_ of the dark-adapted samples, a weak measuring light (<0.5 μmol photons m^−2^ s^−1^) was applied, while for F_m_, an oversaturating light pulse (>10 000 μmol photons m^−2^ s^−1^) was used (Malapascua et al., 2014).

#### 2.4.2. Photometric chlorophyll *a* measurement

Five milliliters of algal biomass at T0 and T3 were filtered onto a Whatman GF/6 glass fiber filter (Ø 25 mm; *n* = 3), and chlorophyll *a* was extracted using 96% ethanol (v/v) and measured spectrophotometrically using the equation given by HELCOM (2019).

To obtain a reference value for the chlorophyll *a* concentration, 5 mL of algal suspension from each culture (*n* = 3) was fixed with Lugol solution at T0 and T3, and the cell number was determined in 1 mL, using a sedimentation chamber at 100x or 200x magnification and an inverted microscope (Olympus IX70, Hamburg, Germany). Always 400 morphologically intact cells were counted, and empty or half-empty valves were ignored. The final amount of cells per mL suspension was calculated as follows:


$$\begin{array}{l} \frac{cells}{mL}=\frac{counted\;cells\;\times D\;\times A\times 1mL}{a\times Sq} \end{array}$$

[D **=** dilution factor; **A** **=** total area of the chamber (mm^2^); Sq **=**amount of counted squares; **a** **=** area of one square (according to used magnification) (mm^2^)].

Chlorophyll *a* measurements were then correlated with the cell counts (*n* = 3 replicates).

#### 2.4.3. Photosynthesis–irradiance curves (P–I curve)

The photosynthetic oxygen production and respiratory oxygen consumption of each strain were determined at 10 different light levels (0–~1,500 μmol photons m^−2^ s^−1^) generated by LEDs (LUXEON Rebel1 LXML-PWN1-0100, neutral-white, Phillips, Amsterdam, Netherlands) implemented into a self-constructed P–I box as described by Prelle et al. (2019). Always, 3 mL of algal suspension was measured in airtight chambers [DW1 oxygen electrode chambers each placed on a magnetic stirrer (Hansatech Instruments, King's Lynn, United Kingdom)] using oxygen dipping probe DP sensors (PreSens Precision Sensing GmbH, Regensburg, Germany) connected to an Oxy 4-mini meter (PreSens Precision Sensing GmbH, Regensburg, Germany) in combination with the PreSens software OXY4v2_30 for measuring and calibration (two-point calibration, 0 and 100% oxygen saturation). The chambers were tempered at 5°C, and 30 μL of sodium bicarbonate (NaHCO_3_, final concentration 2 mM) was added to each sample to avoid carbon deficiency (for details, see Prelle et al., 2019). After each P–I curve, diatom suspension from each cuvette was filtered onto an individual Whatman GF/6 glass fiber filter (Ø 25 mm) for chlorophyll *a* determination as reference parameter (Method see above). The photosynthetic model of Walsby (1997) was used for fitting and calculating different P–I curve parameters.

Since all clonal cultures were not axenic, we estimated the potential influence of bacterial respiration on the diatom net photosynthesis determination. The bacterial volume and the respective diatom volume of T0 samples were determined using DAPI (4′6-diamidine-2-phenylindole) staining. A measure of 500 μL of each glutaraldehyde-fixed T0 sample was filtered on a blackened Polycarbonate Track-Etched Filter (Ø 25 mm, pore size 0.2 μm, Sartorius Lab Instruments GmbH & Co. KG, Goettingen) and stained for 5 min with DAPI (Kapuscinski, 1995). Micrographs of different locations of the filter were taken using an epifluorescence microscope BX-51 (Olympus, Hamburg, Germany) with a 40x lens and CellSens Standard imaging software (Olympus, Hamburg, Germany). For each culture, five micrographs were analyzed using Fiji–ImageJ (version 2.3.0; open source), detecting the area of the blue signal after deleting the shapes of diatoms and determining the area of diatoms in each picture. Additionally, the average diameter of the bacterial cells and the diatoms was measured and used to calculate the volume of both groups. The resulting volume data should, therefore, be regarded as a rough approximation to evaluate the bacterial influence on the oxygen values.

#### 2.4.4. Cell biology

To investigate changes in cell integrity after 3 months of dark incubation, 1 mL of algae suspension was taken (*n* = 3) from T0 and T3 without further fixation and was directly stained with 1 μL of SYTOX Green (Catalog no. S7020, Thermo Fisher Scientific, Waltham, Massachusetts, USA; diluted in culture medium from a 5 mM solution in DMSO). After 5 min of dark incubation, stained and unstained cells were quantified with an epifluorescence microscope (BX-51, Olympus, Hamburg, Germany) under blue excitation (U-MWB, Olympus, Hamburg, Germany). At least 400 unstained cells and the corresponding number of compromised cells were counted in each replicate.

The effects of dark incubation on the volume of lipid droplets were determined using Nile red lipid staining according to Greenspan et al. (1985). Twenty-five cells of each culture at T0 and T3, preferably in the same orientation, were imaged using epifluorescence microscopy (BX-51) under blue excitation (U-MWB) along with a digital camera UC30 and CellSens standard imaging software (all from Olympus, Hamburg, Germany). The width and length of each cell and the respective sizes of lipid droplets were measured with Fiji–ImageJ (version 2.3.0; open source). The cell volume and the volume of a lipid droplet were modulated by an ellipsoid shape. Height and width were assumed to be identical, except for the centric species *Melosira* sp. D323_018, which exhibited numerous lipid droplets. In this study, the number of lipid droplets was counted, and the average diameter of the spherical lipid droplets was determined to calculate the total lipid volume. The cell volume of this culture was idealized and calculated using two hemispheres and a cylinder.

To obtain a more detailed picture of the cell biological changes in darkness, T0 and T3 samples were prepared and fixed by standard chemical fixation for transmission electron microscopy (TEM) according to Holzinger et al. (2009), using 2.5% glutaraldehyde (25% glutaraldehyde diluted with 50 mM cacodylic acid buffer, pH 6.8) and osmium tetroxide (OsO_4_ diluted in cacodylic acid buffer) fixatives. These samples were dehydrated by increasing the alcohol series, embedded in modified Spurr resin according to Holzinger et al. (2009) before being sectioned using an ultra-microtome. Ultrathin sections were stained with uranyl acetate and lead citrate and were investigated with a Zeiss LIBRA 120 transmission electron microscope at 80 kV. Images were captured with a TRS 2k SSCCD camera and further processed using Adobe Photoshop software (Adobe Systems Inc., San José, CA, USA).

#### 2.4.5. GC-MS analysis

For fatty acid analysis of diatom samples at T0 and T3, a gas chromatograph connected to a mass spectrometer (GC-MS) was used according to Schaub et al. (2017). Since this analytical approach is time-consuming, we used only one replicate with three individual injections to identify at least the most conspicuous changes in the main fatty acids. Diatom samples were filtered onto Whatman GF/6 glass fiber filter (Ø 25 mm), freeze-dried and extracted with dichloromethane:methanol (2:1, v/v), and evaporated under nitrogen in a heat block (30°C), and the residue was re-dissolved in dichloromethane:methanol (2:1, v/v) and stored at −20°C until further analysis (Schaub et al., 2017).

For transesterification, 1 mL of lipid extract was evaporated under nitrogen to dryness, dissolved in 250 μL of hexane and heated for 4 h at 80°C with 1 mL of a 3% concentrated sulfuric acid in methanol. Subsequently, fatty acid methyl esters (FAMEs) were extracted three times with hexane, transferred to GC vials, and concentrated under nitrogen down to ~80 μL. GC analysis was carried on a fused silica capillary column (WCOT; 60 m × 0.25 mm I.D.; film thickness 0.25 μm; liquid phase: DB-FFAP; J&W, Germany) with a HP 6890 gas-liquid chromatograph coupled with a 5,970 Series mass selective detector (MSD; Hewlett–Packard GmbH, Germany) and a temperature program according to Kattner and Fricke (1986). The samples were injected at 60°C in splitless mode, with helium as carrier gas. Identification and quantification of fatty acids were according the protocol in Schaub et al. (2017).

#### 2.4.6. Statistics and calculations

All calculations were performed using Microsoft Office Excel (2016). To calculate the P–I curves according to the Walsby model, the solver function was used to minimize the normalized deviation squares. The statistical analysis was performed using SPSS Statistics (version 27). To calculate significance levels among all means, one-way ANOVA was used followed by a *post-hoc* Tukey test. If the data did not fulfill the assumptions of variance homogeneity or normal distribution for one-way ANOVA, the Mann–Whitney test was used in case of two independent groups—in case of three or more independent groups, the Kruskal–Wallis one-factor ANOVA was applied. The significance level was set to α < 0.05 for all analyses.

## 3. Results

### 3.1. Species identification and the description of a new benthic diatom taxon

Five strains from the four sample locations were investigated in this study. The morphological and molecular analyses confirmed the assignment to four distinct species. The taxonomic identity of some strains was already published before (D288_003, D296_001, D300_015; Prelle et al., 2022; Schimani et al., 2023).

D288_003 was identified as *Navicula criophiliforma* Witkowski, Riaux-Gob. and Daniszewska-Kowalczyk. This taxon was first published by Witkowski et al. (2010) from the Kerguelen Islands coastal area, Southern Ocean, and more recently also reported from Livingston Island, north of the Antarctic Peninsula (Zidarova et al., 2022). LM and SEM pictures of this strain can be found in Prelle et al. (2022).

D296_001 was identified as *Chamaepinnularia gerlachei* Van de Vijver and Sterken. This species was first published by Van de Vijver et al. (2010) from dry soil samples from James Ross Island, near the northeastern extremity of the Antarctic Peninsula, and has been observed until now just from maritime Antarctica (Kopalová et al., 2012; Sterken et al., 2015; Zidarova et al., 2016). A thorough examination of this strain can be found in Schimani et al. (2023).

**Figure 2:**
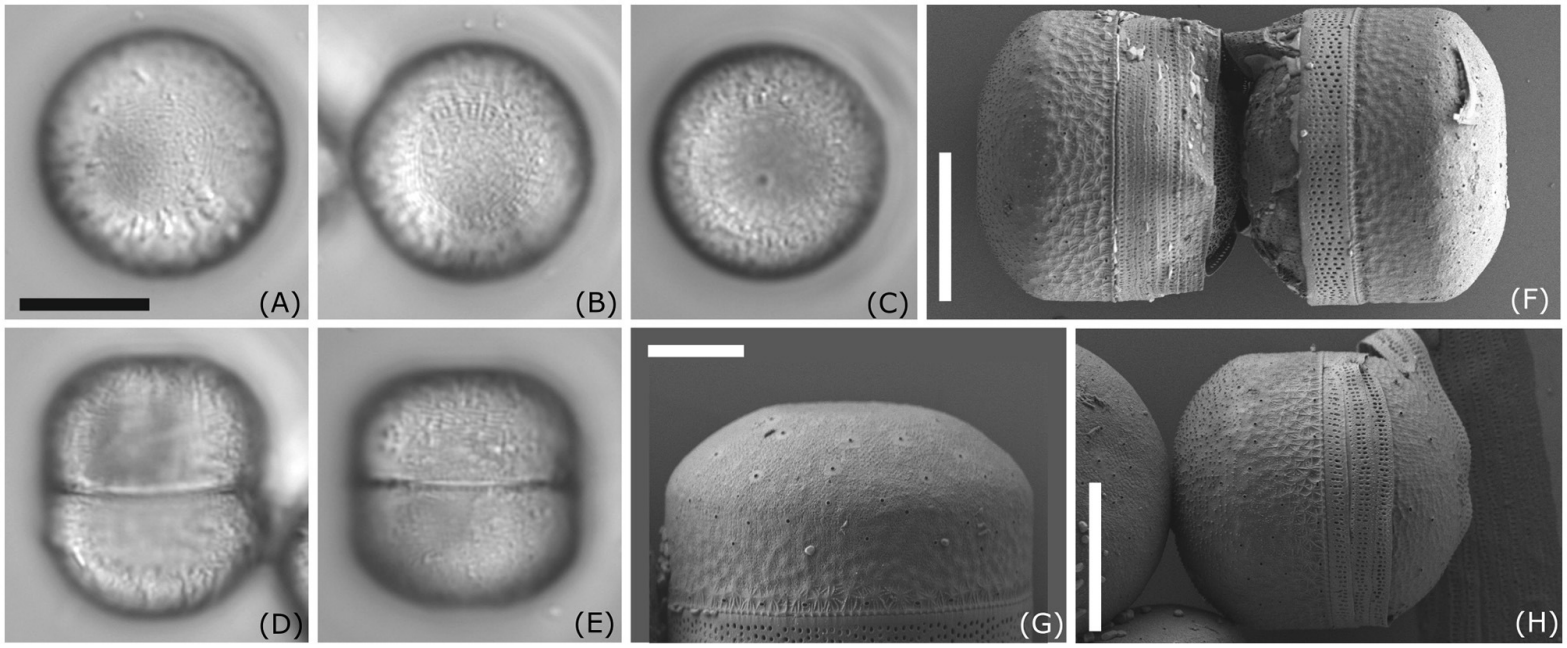
Morphology of *Melosira* sp. D332_018. **(A–E)** Light microscopy images; **(F–H)** scanning electron microscopy images external view; Scale bar: **(A–E, H)** 10 μm; **(G)** 5 μm.

D323_018 (Figure 2) could only be identified at the genus level as *Melosira* sp. Cells are subcylindrical with a diameter ranging between 18.6 and 20.9 μm and a pervalvar axis ranging between 19.4 and 22.0 μm (Figures 2A–E). The hypovalve is more rounded than the epivalve (Figure 2H), which exhibits at the mantel a more cylindrical shape. Our strain resembles *Melosira moniliformis* C. Agardh in Crawford (1977), who examined the type and new material. However, in *M. moniliformis*, ridges on the outer mantle surface fuse to form bigger spines, which are not visible in our strain. Therefore, unambiguous taxonomic assignment of this strain to a particular *Melosira* species is not possible at this stage.

D300_015 was only identified at the genus level as *Planothidium* sp. in Prelle et al. (2022). Morphological and molecular investigations showed that strain D300_025 is the same species. With the new data generated from those strains and two other strains obtained from the same location (D300_019, D300_020), this species is described as new.

***Planothidium wetzelii* Schimani, N. Abarca et R. Jahn sp. nov**.

**Description:**

**Figure 3:**
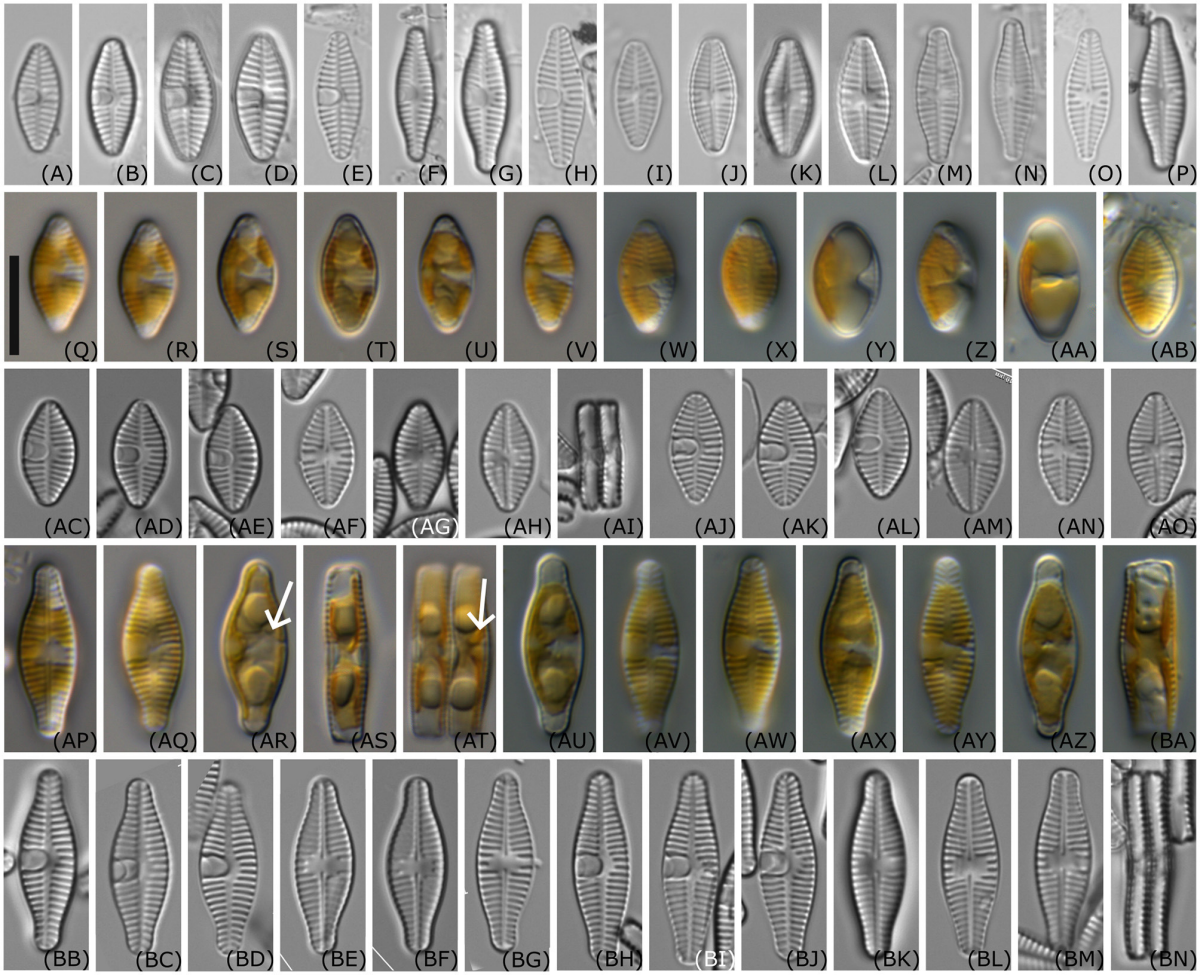
Light microscopy images of *Planothidium wetzelii* sp. nov. **(A–P)** valves of environmental samples; **(Q–V)** live material of strain D300_015; **(W–AB)** live material of strain D300_019; **(AC–AI)** oxidized material of strain D300_015; **(AJ–AO)** oxidized material of strain D300_019; **(AP–AT)** live material of strain D300_020, **(AR, AT)** arrows indicate possible location of one pyrenoid in vicinity of the cavum; **(AU–BA)** live material of strain D300_025; **(BB–BG)** oxidized material of D300_020; **(BH–BN)** oxidized material of strain D300_025; Scale bar: 10 μm.

Living material (Figures 3Q–AB, AP–BA): One plate of the C-shaped plastid lies appressed to the valve face. The concavity of the plastid is visible on the valve side lacking the cavum. Pyrenoids are not clearly visible, but Figures 3AR, AT suggest that there is one pyrenoid located in the vicinity of the cavum. In the girdle view, especially in the larger cells before cell division (Figure 3BA), two lobes of the chloroplast are visible appressed to the valves, and connected in the center of the cell.

LM (Figures 3A–P, AC–AO, BB–BN): The outline ranged from elliptic with slightly protracted rostrate apices in smaller valves to elliptic or lanceolate with protracted, rostrate to capitate apices in larger valves. One valve side—especially strain D300_020—appeared more convex than the other. The length of the valves varied between 10.9 and 18.8 μm and the width between 5.6 and 6.7 μm (*n* = 43). Sternum valve (SV): Axial area narrow, becoming wider toward the center of the valve. Central area asymmetrical with an oblong cavum with parallel sides, extending slightly over the axial area on one side and with shortened striae on the other side. The parallel margins of the cavum widen just shortly before the mantle, and its aperture is seen as a roundish line close to the mantle. Striae parallel or weakly radiating in the center, becoming more radiate near the apices (16–18 in 10 μm in smaller valves and 14–16 in 10 μm in larger valves). Raphe valve (RV): Axial area narrow. The central area widened due to three to four shortened striae on both sides. Raphe straight with enlarged proximal endings. Distal endings are not visible in light microscopy. Striae are parallel or weakly radiating in the center, becoming more radiating near the apices (16–18 in 10 μm in smaller valves and 14–15 in 10 μm in larger valves).

**Figure 4:**
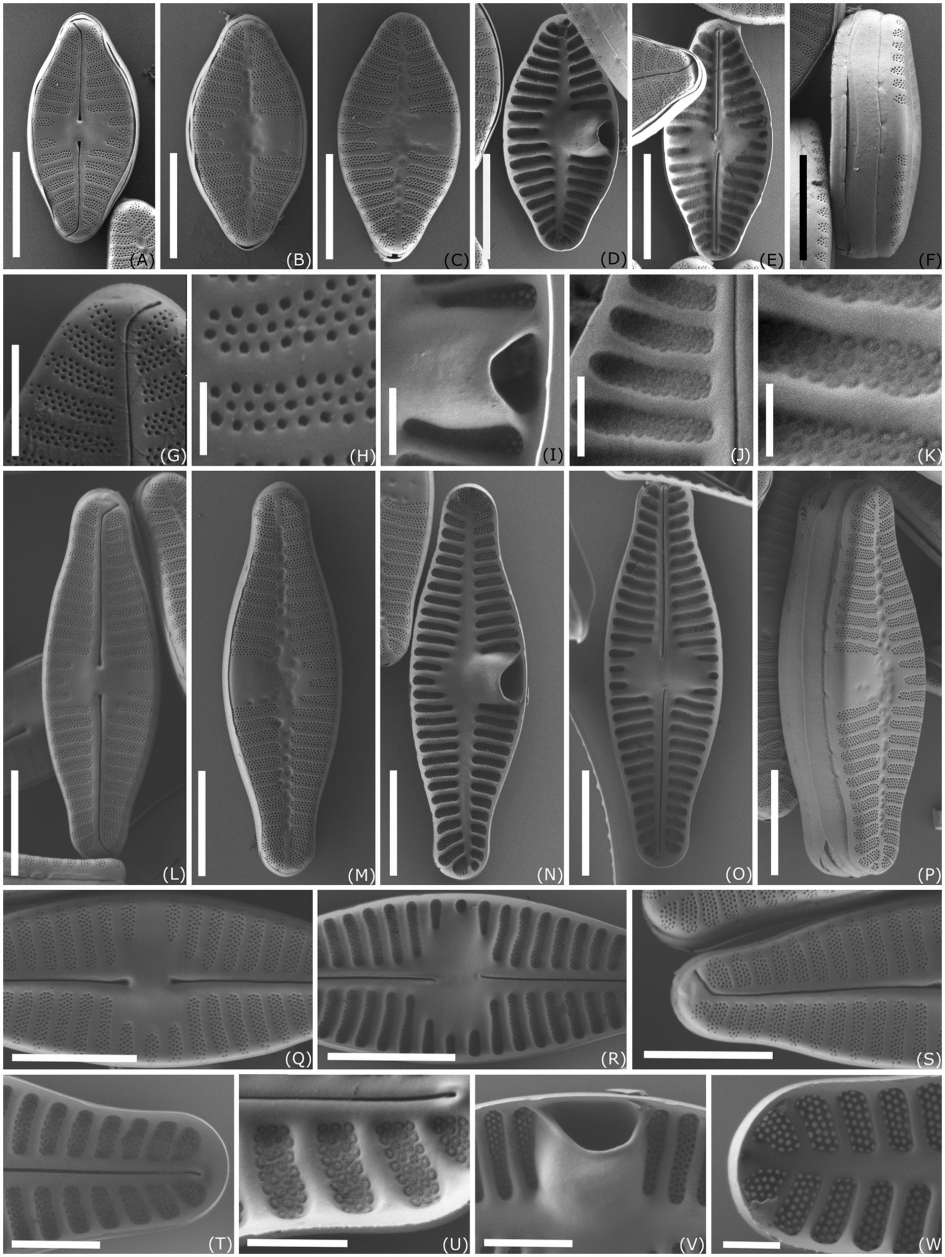
Scanning electron microscopy images of *Planothidium wetzelii* sp. nov. **(A–K)** strain D300_015; **(L–W)** strain D300_025; **(A, E, G, H, J–L, O–T)** raphe valve (RV); **(B–D, F, I, M, N, P, V, W)** sternum valve (SV); **(A–C, F–H, L, M, P, Q, S)** external view; **(J, K, U)** internal view of RV, showing three to four rows of same sized hymenate areolae in one stria; **(I, V)** internal view of central area in RLV showing cavum; Scale bar: **(A–F, L–P)** 5 μm; **(Q–S)** 4 μm; **(G, T, V)** 2 μm; **(I, J, U, W)** 1 μm; **(H, K)** 0.5 μm.

SEM (Figures 4A–W) SV: The striae are composed of three to four rows of small rounded areolae exceeding the width of the virgae. Striae near the center of the valve become pointed with one or two areolae near the axial area. Externally, striae reach over the mantle with up to four rows of areolae. Irregularly rounded depressions are externally present along the axial area and concentrated in the central area. Internally, areolae are covered by hymenate occlusions. The broad cavum had a tight hood opening close to the mantle. Cingulum is composed of unperforated girdle bands. RV: The striae are composed of usually three to four rows of rounded areolae exceeding the width of the virgae. Similar to the central striae on the SV, the shortened central striae on the RV gradually narrow toward the axial area. At each stria, one to three areolae reach over the edge of the valve face. Internally, areolae are covered with the same hymenate occlusions as in SV. Externally, the raphe straight with drop-like expanded proximal ends and distal raphe fissures unilaterally deflected, not or just shortly continuing onto the valve mantle. Internally, proximal raphe endings are slightly bent to opposite sides, and raphe endings terminate on a small helictoglossae not continuing on the valve mantle.

**Holotype:** Slide B 40 0045341a, Botanic Garden and Botanical Museum, Berlin, Figure 3BJ for SV and Figure 3BK for RV from the strain D300_025 illustrate the holotype. SEM-stub deposited as B 40 0045341b. For molecular material and data, see Methods.

**Type locality:** Drinking water reservoir of Carlini Station, Potter Cove, King George Island, South Shetland Islands, collected by J. Zimmermann on 01 February 2020, coordinates: S 62.237861, W 58.662250.

**Registration:** http://phycobank.org/103793.

For INSDC Accession numbers see Table 1.

**Habitat:** Freshwater biofilm on stones.

**Etymology:** With this name, we would like to acknowledge the morphological and taxonomic work on the genus *Planothidium* done by Carlos Wetzel (Luxembourg Institute of Science and Technology, Luxembourg) in recent years, as well as his help in finding the identity of this species.

**Differential diagnosis:** *Planothidium wetzelii* shares similarities with several cavum-bearing species and species with external shallow rounded depressions on the rapheless valve (as defined by Wetzel et al., 2019): The smaller valves of *P. wetzelii* share similarities in shape with the small valves of *P. frequentissimum* (Lange-Bertalot), Lange-Bertalot as illustrated from type material in Wetzel et al. (2019). However, they can be distinguished by the form of the cavum, which is oblong with parallel sides and extending slightly over the axial area, vs. a round cavum in *P. frequentissimum*. The shape of the central area on the RV is much smaller in *P. wetzelii* compared to the type of *P. frequentissimum*. In SEM, the virgae in the SL and to some extent on the RV are wider in *P. wetzelii* compared to images of the type of *P. frequentissimum*. Additionally, the striae on the RV do not continue over the mantle in *P. frequentissimum* as they do in *P. wetzelii*. Larger valves of *P. frequentissimum* are lanceolate with less developed rostrate apices than those of *P. wetzelii*. Valves of *P. victorii* Novis, Braidwood et Kilroy (syn. *P. caputium*) are in contrast to *P. wetzelii* lanceolate to weakly elliptic-lanceolate with protracted but never capitate apices (Jahn et al., 2017; Wetzel et al., 2019). The species can be differentiated by the form of the cavum too as it is rounder or has a V-shape in *P. victorii*. In addition, the tight hood opening in *P. wetzelii* (SEM), seen as a roundish line close to the mantle (LM), differs from the wider hood opening (SEM) in *P. victorii*, which is present as an almost straight line distant from the mantle (LM). The axial area is narrower in this species and the cavum has a wider aperture toward the mantle than *P. wetzelii*. Some of the medium sized valves could be confused with *P. naradoense* R.Jahn et J.Zimmermann but differ from *P. wetzelii* by the outline, as *P. naradoense* is lanceolate to elliptic-lanceolate and somewhat asymmetric with slightly rostrate and rounded apices. The axial area is narrow on both valves (Jahn et al., 2017). Larger valves of *P. wetzelii* share similarities with *P. tujii* and *P. gallicum* C. E. Wetzel et Ector (Wetzel et al., 2019). However, the valves of *P. tujii* are shorter, while the width is almost in the same range. The central area in the RV of *P. tujii* is bowtie-shaped instead of the smaller rounded central area in *P. wetzelii*. The virgae are wider in *P. tujii* and have almost the same width as the striae; the helictoglossa is continuing onto the valve mantle. *P. gallicum* has clear broadly elliptic-lanceolate shaped valves with markedly rostrate apices. Furthermore, *P. gallicum* has a more prominent aperture of the cavum toward the mantle, and the striae located in the central area are not becoming narrower toward the axial areal as in *P. wetzelii*. *P. straubianum* C. E. Wetzel, Van de Vijver et Ector has elliptic-lanceolate valves with slightly parallel margins and round, obtuse ends with smaller dimensions than *P. wetzelii* (length 6.0–14.0 μm vs. 10.9–18.8 μm and width 4.0–5.5 μm vs. 5.6–6.7 μm; Wetzel et al., 2019). Striae are composed of four to five rows of areolae, in contrast to three to four in *P. wetzelii*. *P. biporomum* (M. H. Hohn et Hellerman) Lange-Bertalot can be differentiated from *P. wetzelii* in SEM by the striae on the rapheless valve (Wetzel et al., 2013). The striae start externally with one, rarely two, areolae at the axial area and end in two or three rows toward the valve face/mantle junction. Additionally, they are interrupted at the junction with the valve mantle. Furthermore, the cavum aperture is wide with its borders linked to the neighboring striae. *P. alekseevae* Gogorev et E. K. Lange has smaller dimensions (length 9.5–13.5 μm, width 4.5–5.0 μm) and wider apices in relation to the valve width. This feature is more apparent in smaller valves (Wetzel et al., 2019).

**Molecular results:** The four strains of *P. wetzelii* showed no intraspecific variability in the 18S V4 and the *rbc*L sequences (Supplementary Tables 1, 2). Compared to the available sequences of other *Planothidium* species, genetic distances are apparent in both marker genes. It is placed within the F2 subclade (Jahn et al., 2017, p. 84, Figure 1): strains of *P. victorii* (e.g., type strain from New Zealand), *P. straubianum* (strain B86_3 from Lake Baikal, renamed by Wetzel et al., 2019), and *P. tujii* show the smallest genetic divergence: in 18S V4 0.5–1.1, 0.7–0.9, and 0.9–1.1%, respectively, which corresponds to 3–5 bp differences, and in *rbc*L 0.5–0.8, 0.5, and 0.5% (3–7 bp). Higher genetic differences are evident for *P. naradoense* and *P. frequentissimum*: in 18S V4 2.5% and 2.5–3.2% (11 bp) and in *rbc*L 2.2 and 1.8–2.3%, respectively (8–22 bp). Species with a sinus instead of a cavum [*P. lanceolatum* (Brébisson ex Kützing) Lange-Bertalot, *P*. cf. *subantarcticum, P. taeansa* R. Jahn and N. Abarca, *P. cryptolanceolatum* R. Jahn and N. Abarca, and *P. suncheonmanense* R. Jahn and J. Zimmermann] exhibit the highest divergence with a p-distance of 4.3–7.8% (19–38 bp) in 18S V4 and 3.0–6.2% (30–61 bp) in *rbc*L.

### 3.2. Photosynthesis and respiration

The values for Y(II)_max_ at T0 and T3 are shown in Table 2 (mean values ± SD, *n* = 3). All results were between 0.53 (*P. wetzelii* (D300_025) at T3) and 0.63 (*N. criophiliforma* and *C. gerlachei* at T0) and therefore within a range reflecting “good” physiological activity of all diatom cells during the dark incubation. Small, but significant differences in Y(II)_max_ between T0 and T3 were found only in both cultures of the limnic species *P. wetzelii* (Table 2).

**Table 2:** Results of different photosynthesis-related measurements before dark incubation (T0) and after 3 months of dark incubation (T3) of five Antarctic benthic diatom species.

**Culture**	**Treatment**	***F*_v_/*F*_m_**	**Chl a per cell**	**NPPmax**	**Respiration**	**NPP_max_: respiration**
			**(ng Chl per cell)**	**(**μ**mol O**_2_ **mg**^−1^ **Chl** ***a*** **h**^−1^**)**	**(**μ**mol O**_2_ **mg**^−1^ **Chl** ***a*** **h**^−1^**)**	
*Navicula criophiliforma*	T0	0.63 ± 0.01	8.18 ± 8.86	80.0 ± 20.2	−72.2 ± 70.3	2.1 ± 1.5
		*a*	*a*	*a*	*a*	*a*
	T3	0.6 ± 0.02	2.21 ± 1.67	76.9 ± 8.4	−35.8 ± 14.5	2.3 ± 0.6
		*a*	*a*	*a*	*a*	*a*
*Chamaepinnularia gerlachei*	T0	0.63 ± 0.01	0.43 ± 0.51	33.9 ± 2.2	–30.9 ± 10.0	1.2 ± 0.4
		*a*	*a*	*a*	*a*	*a*
	T3	0.57 ± 0.02	0.13 ± 0.02	20.4 ± 2.9	–26.3 ± 1.8	0.8 ± 0.1
		*a*	*b*	*b*	*a*	*a*
*Melosira sp*.	T0	0.57 ± 0.01	4.16 ± 2.69	76.1 ± 9.4	−16 ± 8.4	6.6 ± 5.0
		*a*	*a*	*a*	*a*	*a*
	T3	0.55 ± 0	2.22 ± 1.57	42.2 ± 7.2	−9.4 ± 2.9	5.0 ± 2.2
		*a*	*b*	*b*	*a*	*a*
*Planothidium wetzelii* (D300_015)	T0	0.61 ± 0	0.93 ± 0.08	16.7 ± 4.0	−7.8 ± 6.2	9.0 ± 14.0
		*a*	*a*	*a*	*a*	*a*
	T3	0.57 ± 0.01	0.95 ± 0.4	13.4 ± 4.7	−15.4 ± 2.4	0.9 ± 0.4
		*b*	*a*	*a*	*a*	*a*
*Planothidium wetzelii* (D300_025)	T0	0.57 ± 0.03	1.53 ± 1.37	39.6 ± 9.8	−31.0 ± 4.3	1.3 ± 0.5
		*a*	*a*	*a*	*a*	*a*
	T3	0.53 ± 0	1.345 ± 0.56	25.3 ± 11.2	−30.5 ± 19	1.2 ± 0.8
		*b*	*b*	*a*	*a*	*a*

Maximum quantum yield (*F*_v_/ F_m_) of PS II measured by pulse amplitude modulation (PAM) fluorimetry. Data are shown as mean ± standard deviation (n = 3). Chlorophyll a content is given as ng Chl a per cell (n = 3). NPP_max_ represents the maximum net primary production rate derived from P–I curves in the PI box at 5°C (n = 4, D300_025 n = 3). Different lowercase letters (a, b) represent significance levels among all means as calculated by one-way ANOVA (Tukey's test, *p* < 0.05). Significance concerning NPP_max_, respiration, and NPP_max_: Respiration was analyzed by Kruskal–Wallis analysis (*p* < 0.05) due to non-normally distributed data points.

The results of the chlorophyll *a* measurements, which were referenced to cell counts, showed species-specific responses (Table 2). In both strains of *P. wetzelii*, the chlorophyll *a* content per cell did not significantly change during 3 months of dark treatment. In contrast, *N. criophiliforma* and *C. gerlachei* exhibited a pronounced chlorophyll *a* decline up to 74% after dark incubation compared to the control, while the *Melosira sp*. decreased chlorophyll *a* by ~46% (Table 2).

All measured P–I curves exhibited a typical shape without photoinhibition, as already reported for some of the species in detail in Prelle et al. (2022). The key parameters NPP_max_ and respiration were selected for all species before and after dark incubation (Table 2), and species-specific responses could be outlined. At T0, the highest value for NPP_max_ was determined in the marine species *N. criophiliforma* (80.0 ± 20.2 μmol O_2_ mg^−1^ Chl *a* h^−1^), while *P. wetzelii* (D200_015) showed the lowest NPP_max_ (16.7 ± 4.0 μmol O_2_ mg^−1^ Chl *a* h^−1^). While some species (*N. criophiliforma*, both *P. wetzelii* isolates) did not exhibit any significant NPP_max_ decline (*p* < 0.05) during dark treatment at T3, *C. gerlachei* and *Melosira* sp. showed a strong and significant decrease (*p* < 0.05) in NPP_max_ by 39.9 and 44.6%, respectively (Table 2).

Although respiration rates were also species-specifically different and variable, we could not detect any significant difference in respiration between T0 and T3 among all benthic diatom species (Table 2). While the highest respiration values occurred in the marine species *N. criophiliforma* (−72.2 ± 70.3 O_2_ mg^−1^ Chl *a* h^−1^), the lowest respiration rates were measured in the limnic culture *P. wetzelii* (D300_015, −7.8 ± 6.2 O_2_ mg^−1^ Chl *a* h^−1^). The NPP_max_: Respiration ratios were relatively low for all isolates between 0.8 ± 0.1 and 2.9 ± 4.0 at T0 and T3, except for *Melosira* sp. which exhibited higher ratios of 6.6 ± 5.0 and 5.0 ± 2.2, respectively, due to proportionally lower respiration rates (Table 2). All diatom cultures were not axenic, and the bacterial abundance ranged from a very low 1.7% bacteria volume when compared with the diatom volume in *Melosira* sp. to 23.7% in *P. wetzelii* (D300_25; Table 2). All other species showed bacterial contamination between these extreme percentages.

### 3.3. Membrane integrity and ultrastructure

**Figure 5:**
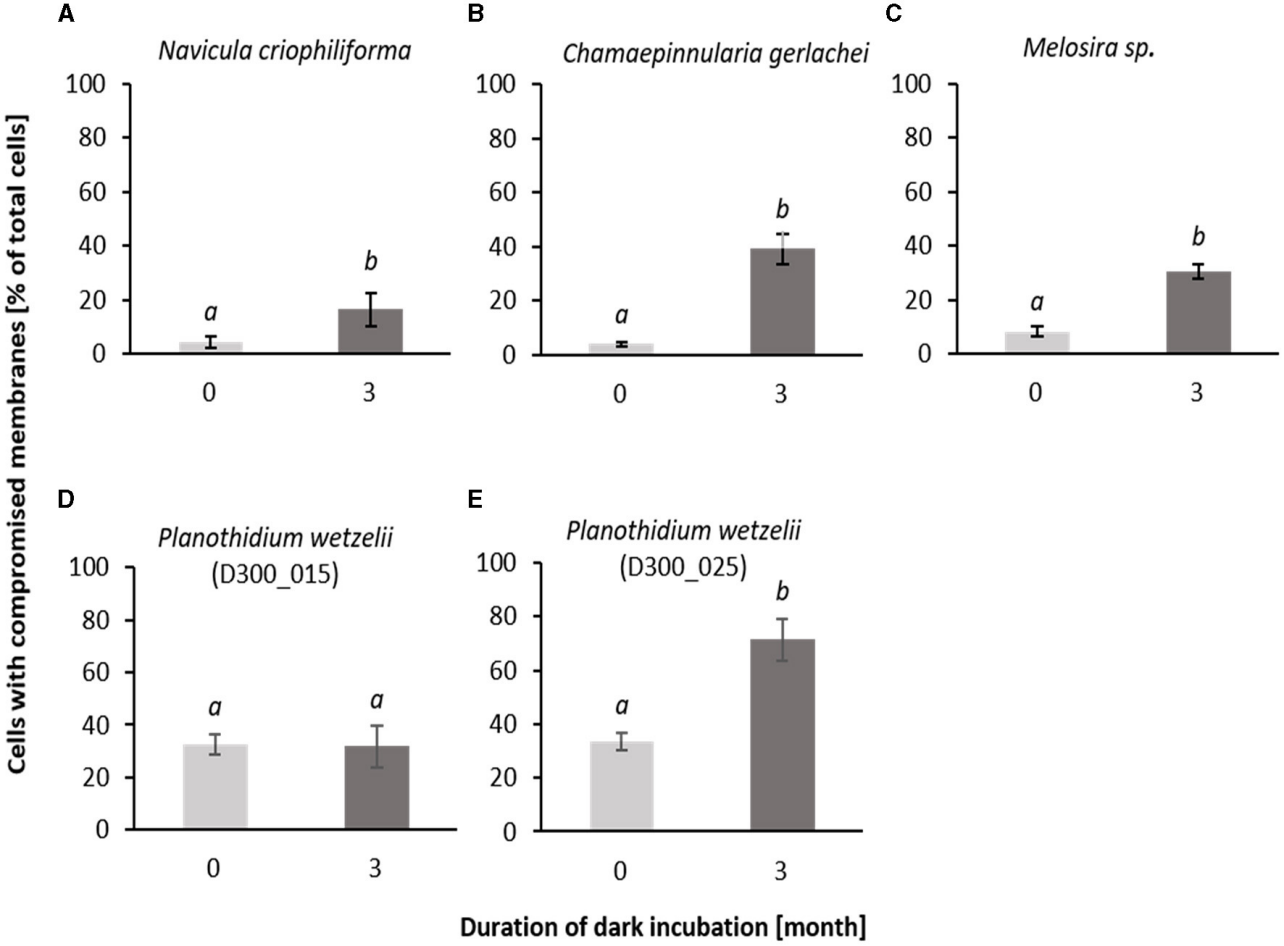
Stained cells of all investigated Antarctic benthic diatoms **(A–E)** with SYTOX Green as the percentage of total counted cells (stained and not stained) before and after 3 months of dark incubation. 400–700 cells were counted for each treatment and taxon. Data are shown as mean ± standard deviation (*n* = 3). Different lower case letters (*a* and *b*) represent significance differences calculated by one-way ANOVA (Tukey's test, *p* < 0.05).

SYTOX Green staining was applied as a cell biological approach to evaluate membrane integrity during dark incubation. The percentage of damaged cells increased significantly in four out of the five tested benthic diatom species (Figure 5). In *N. criophiliforma, C. gerlachei*, and *Melosira* sp. at T0 only <5% of the cells were damaged pointing to highly viable cell populations. After 3 months, dark incubation membrane integrity decreased in all three species, i.e., cells with compromised membranes accounted for between 16.4 and 39.0% (Figure 5). Both *P. wetzelii* strains were already at T0 and less viable as reflected in approximately one-third of the damaged cells. While isolate D300_025 exhibited 71.5% compromised membranes, *P. wetzelii* (D300_015) maintained membrane integrity over 3 months of dark incubation (Figure 5).

**Figure 6:**
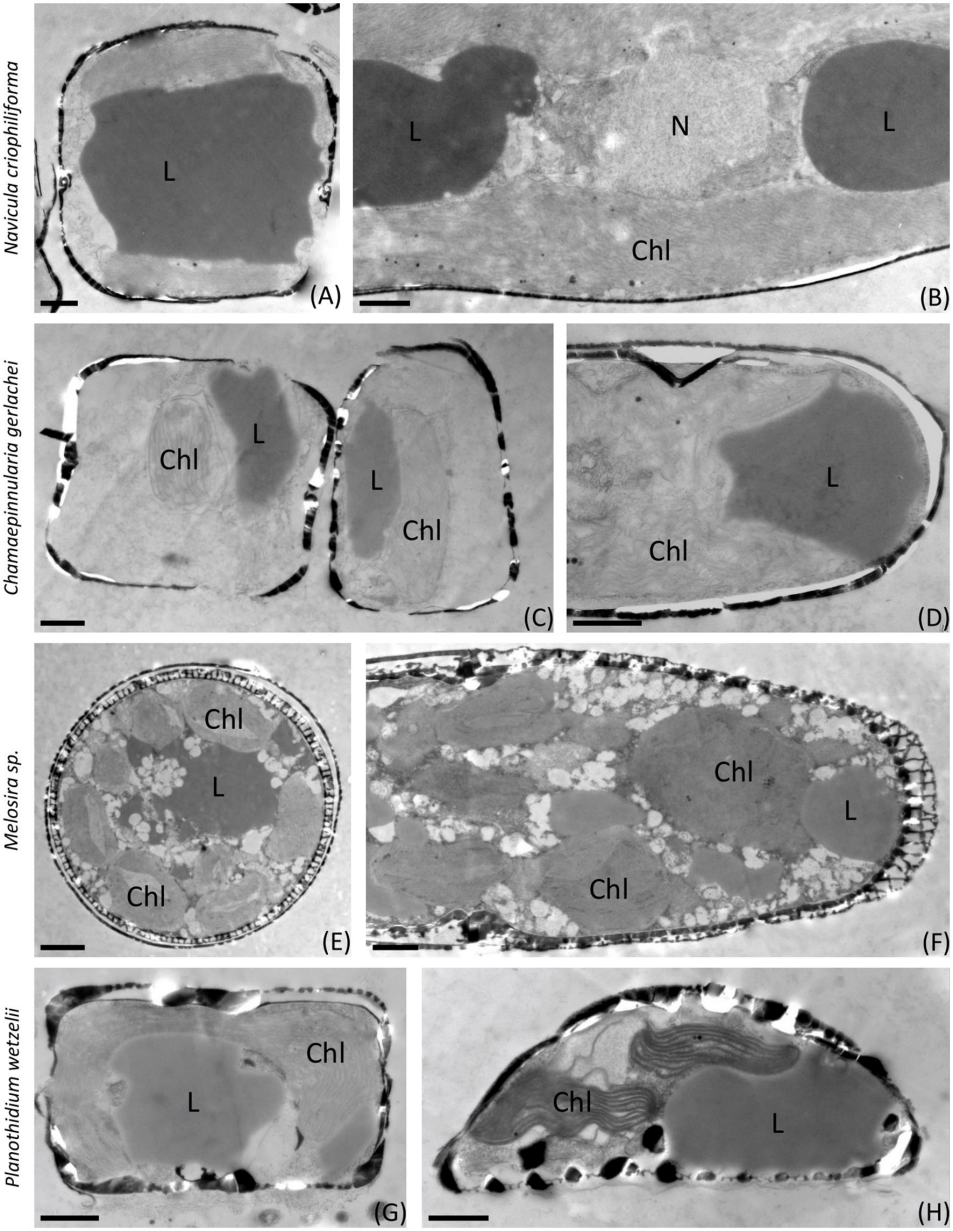
Transmission electron micrographs of control samples (T0). **(A, B)** *Navicula criophiliforma*, massive lipid droplets on both sides of the nucleus, intact chloroplast with thylakoid membranes; **(C, D)** *Chamaepinnularia gerlachei*, lipid droplets and intact chloroplasts; **(E, F)** *Melosira* sp., several small chloroplasts visible, lipid droplets and small electron translucent particles in the cell lumen, characteristic cell wall pattern; **(G, H)** *Planothidium wetzelii* (D300_015). Chl, chloroplast; L, lipid droplet; N, nucleus. Scale bars: 1 μm.

**Figure 7:**
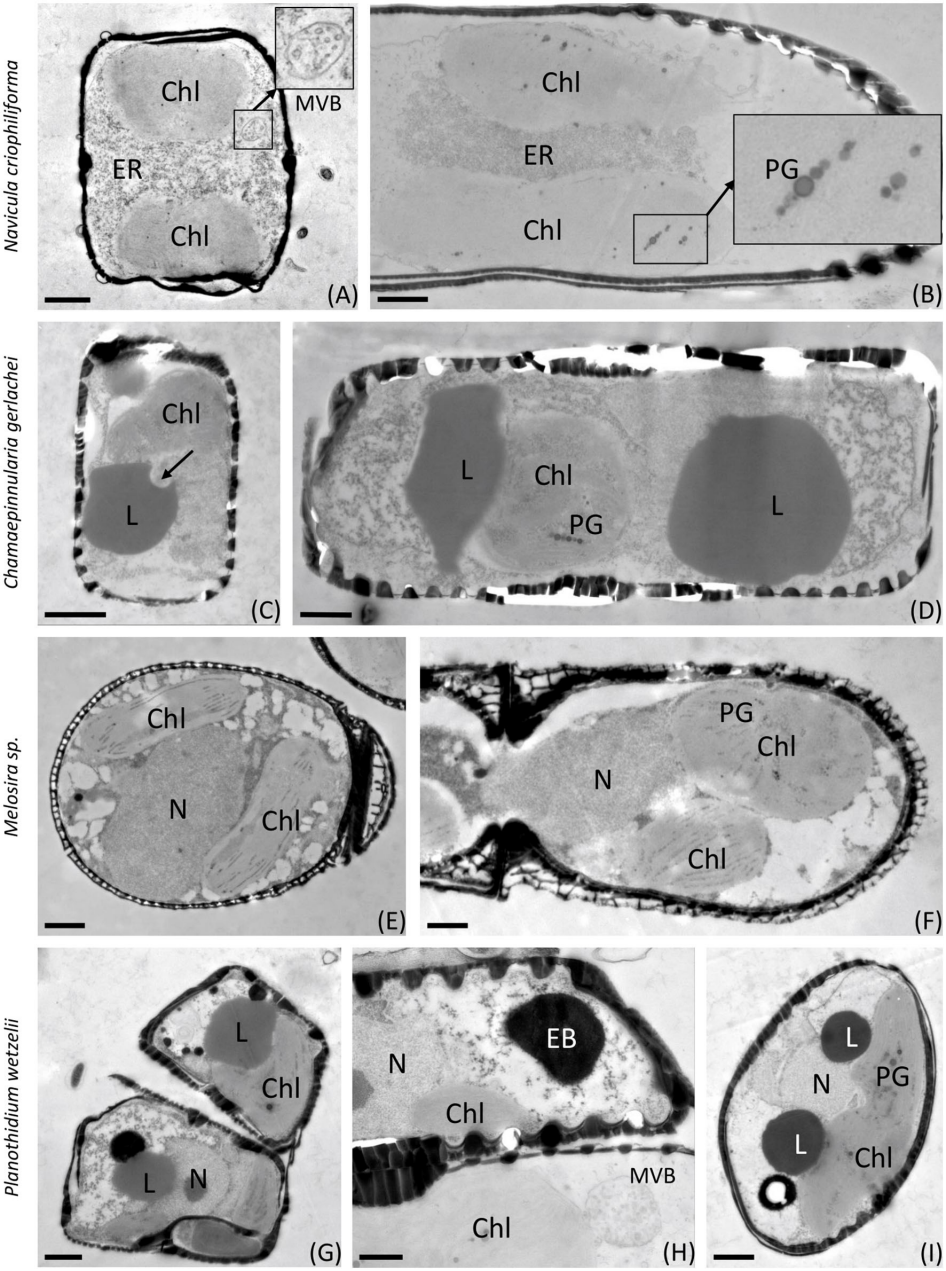
Transmission electron micrographs of 3 months of dark-treated samples (T3). **(A, B)** *Navicula criophiliforma*, chloroplasts are virtually lacking thylakoids, rows of plastoglobules are visible, massive accumulations of ER in the cell center, no lipid droplets are visible; **(C, D)** *Chamaepinnularia gerlachei*, lipid droplets are visible, and signs of lipid degradation are marked with an arrow; **(E, F)** *Melosira* sp., chloroplasts with reduced thylakoid membranes, containing plastoglobules; **(G–I)** *Planothidium wetzelii* (D300_015), chloroplasts reduced, lipid droplets visible, sometimes in close contact with electron-dense bodies. Chl, chloroplast; EB, electron-dense body; ER, endoplasmic reticulum; L, lipid droplet; MVB, multivesicular body; N, nucleus; PG, plastoglobules. Scale bars: 1 μm; (insets) 250 nm.

Figures 6, 7 show the first transmission electron micrographs (TEMs) of benthic diatom species from Antarctica under control (T0, Figure 6) conditions and after 3 months of dark incubation (T3, Figure 7). The three pennate (*N. criophiliforma, C. gerlachei*, and *P. wetzelii*) and one-centric diatom species (*Melosira* sp.) exhibited the typical cellular ultrastructure of a diatom cell. All cells contained a central nucleus, several chloroplasts, and, most conspicuously, one or more large lipid droplets that occupied large parts of the cell volume (Figure 6). After the dark treatment, ultrastructural changes could be detected (Figure 7). The most conspicuous observations were degraded chloroplasts with plastoglobules (PGs) in all species and partially more endoplasmic reticulum (ER). In addition, the volume or number of lipid droplets drastically decreased (Figure 7). In *N. criophiliforma*, lipid droplets were virtually absent (Figures 7A, B) and only observed in a few cases. Similarly, in *Melosira* sp., no or only very small lipid droplets occurred at T3. In contrast, in *C. gerlachei* and *P. wetzelii* (D300_015), well-developed lipid droplets could be identified. However, in Figure 7C of *C. gerlachei* beginning, the degradation of a lipid droplet was observed (marked by an arrow). In addition, *N. criophiliforma* and *P. wetzelii* (D300_015) exhibited multivesicular bodies (MVBs) after 3 months of darkness (Figures 7A, H), a typical sign of apoptosis. Additionally, in *P. wetzelii*, several larger electron-dense bodies were observed (Figures 7G, H). While the origin of these bodies remains unknown, they might resemble the degradation products of the chloroplasts.

### 3.4. Lipid volume and composition

**Figure 8:**
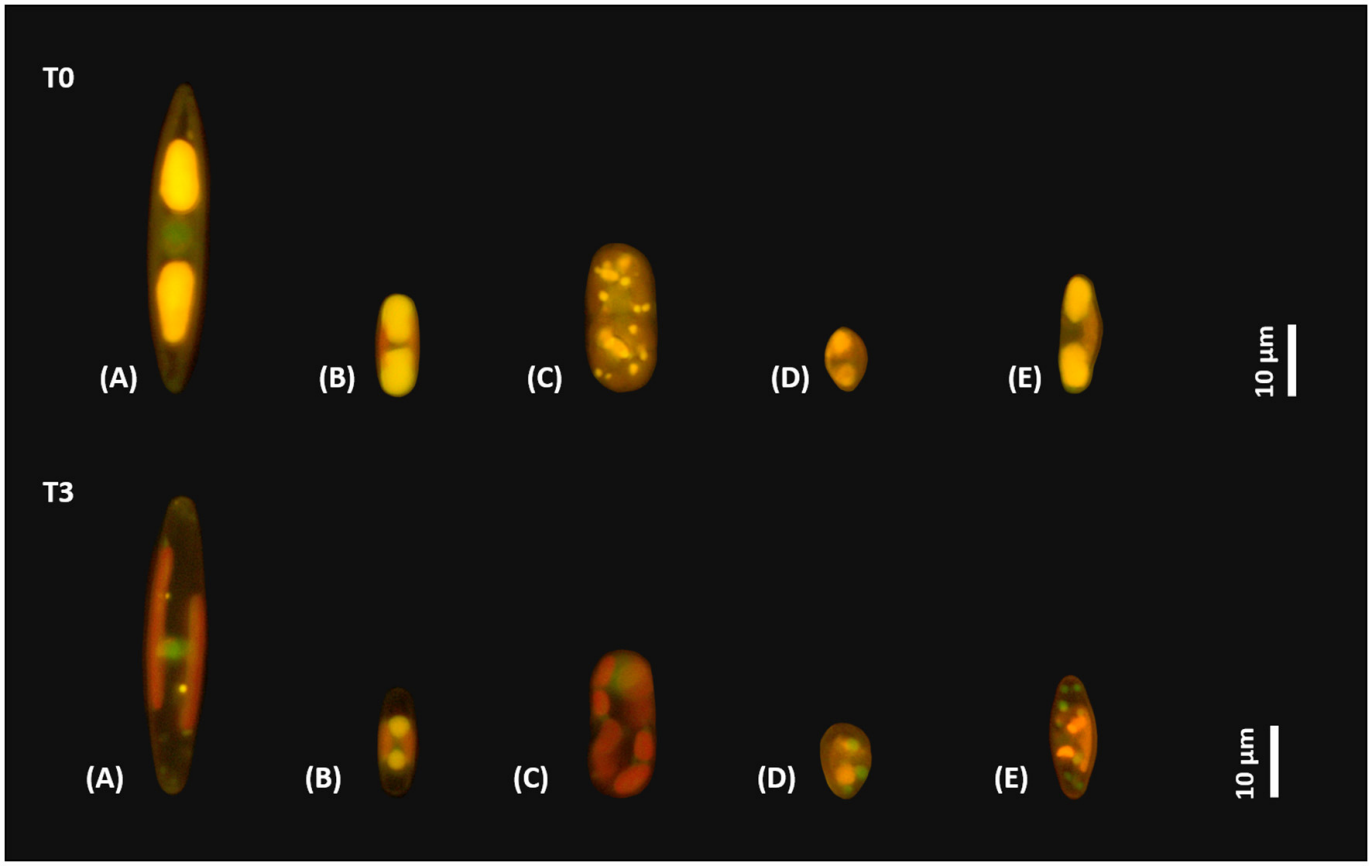
Micrographs of five Antarctic benthic diatom strains stained with Nile red. Upper row shows lipid droplets of the control, and lower row shows lipid droplets after 3 months of dark incubation. **(A)** *Navicula criophiliforma*; **(B)** *Chamaepinnularia gerlachei*; **(C)** *Melosira* sp.; **(D)** *Planothidium wetzelii* (D300_015); **(E)** *Planothidium wetzelii* (D300_025).

**Figure 9:**
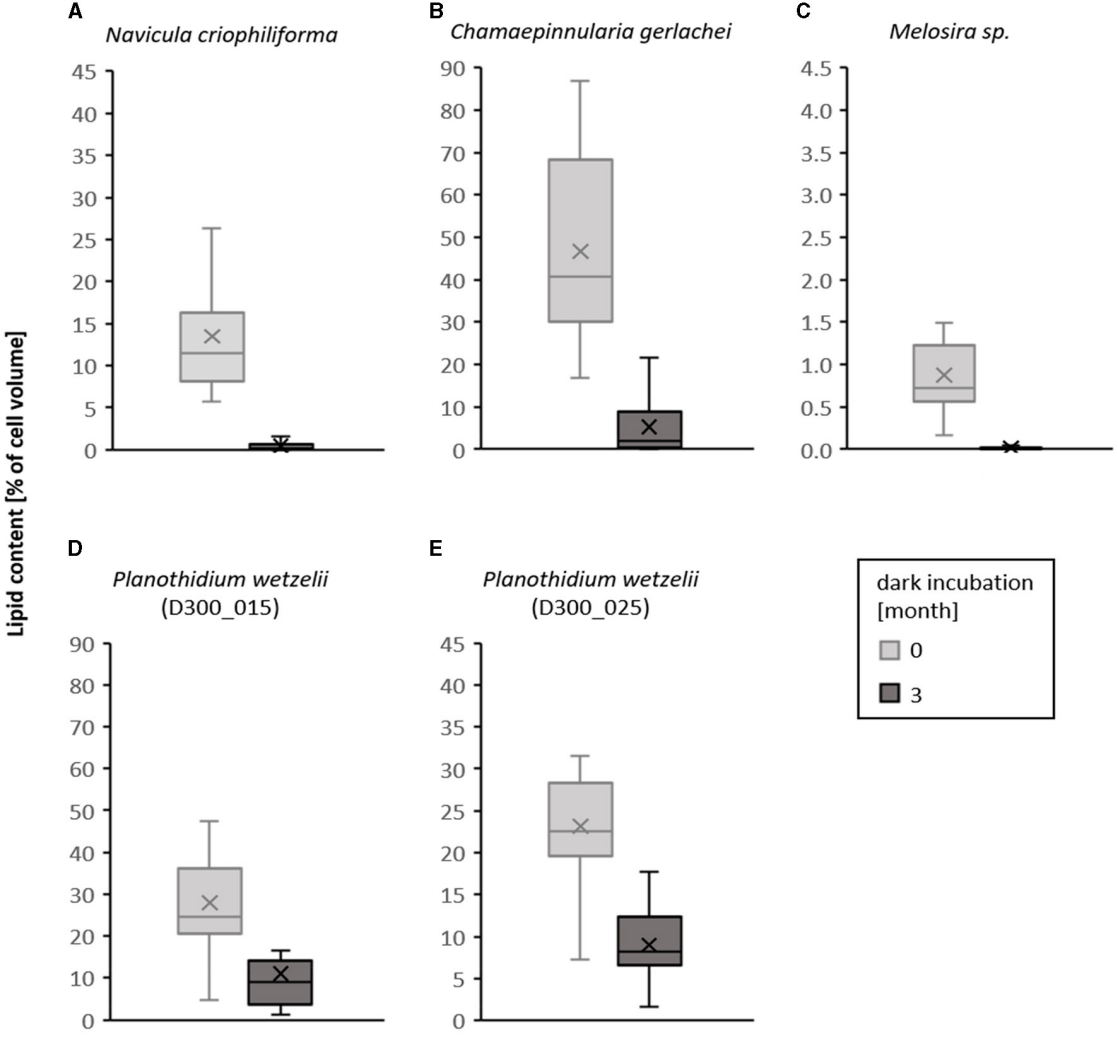
Boxplots of lipid content per cell calculated as percentage cell volume before dark incubation (light gray) and after 3 months of dark incubation (dark gray) in five Antarctic benthic diatom strains **(A–E)**. For each culture and time, 25 cells were measured. Scaling of the Y-axis is proportional but different as the lipid content differs. Different lower case letters represent significance differences calculated by Mann–Whitney *U*-test (*p* < 0.05).

The visual differences of the species-specific lipid content after Nile red staining of the diatom cells before and after 3 months of dark incubation are summarized in Figure 8. In this study, a clear decrease in the size of the stained lipid droplets can be seen. The corresponding calculated lipid volume changes in the examined diatom cells are shown in Figure 9 as boxplots of 25 measured cells each. A significant decrease in the lipid content over 3 months of dark incubation could be documented for all species. The marine isolates *N. criophiliforma, C. gerlachei*, and *Melosira* sp. exhibited >95% the strongest decrease in the total lipid content per cell within 3 months of darkness, and in the first and third species only traces of lipids remained detectable (Figure 9). In contrast, in both *P. wetzelii* strains, the lipid content changed much less, with a decrease to only 58.7–60.7% of the control, i.e., these cells contained at T3 still detectable amounts of lipids.

The chemical GC-MS analysis of the total lipid content supports the results shown in Figures 8, 9, i.e., the marine isolates *N. criophiliforma, C. gerlachei*, and *Melosira* sp. exhibited concentration declines between 90.0 and 94.5% (Table 3). In both limnic *P. wetzelii* strains, the lipid content decreased by 20.6 and 35.2% after 3 months of darkness (Table 3). Saturated fatty acids (SFAs), monounsaturated fatty acids (MUFAs), and polyunsaturated fatty acids (PUFAs) before and after 3 months of dark incubation were also evaluated, but a clear trend is not visible, since all species showed different response patterns. The quantitatively most abundant fatty acids in all benthic diatoms were the saturated 16:0 and the monosaturated 16:1n7 (Table 3). While the percentage proportion of 16:0 in relation to all measured fatty acids increased in *N. criophiliforma* (from 43.6 to 58.4%) and *C. gerlachei* (from 38.3 to 48.1%) after 3 months of darkness, it strongly decreased in the remaining species (e.g., in *Melosira* sp. from 36.2 to 16.9%). Reversely, the percentage proportion of 16:1n7 in relation to all fatty acids strongly declined in *N. criophiliforma, C. gerlachei*, and *Melosira* sp., while both *P. wetzelii* strains exhibited a slight increase (Table 3).

**Table 3:** Lipid analysis using gas chromatography connected to a mass spectrometer (GC-MS).

**Dark incubation [months]**	***Navicula criophiliforma***		***Chamaepinnularia gerlachei***		***Melosira*** **sp**.		***Planothidium wetzelii*** **(D300_015)**		***Planothidium wetzelii*** **(D300_025)**	
	**T0**	**T3**	**T0**	**T3**	**T0**	**T3**	**T0**	**T3**	**T0**	**T3**
Total lipid content [pg/cell]	43.6 ± 59.5	2.4 ± 1.2	9.6 ± 6.1	1.2 ± 0.3	27.8 ± 17.3	2.8 ± 2.2	6.5 ± 2.5	4.2 ± 1.7	21.9 ± 6.6	17.4 ± 4.8
Decrease in total lipid content [%]	−94.5		−87.6		−90		−35.2		−20.6	
Saturated fatty acids (SFAs) [%]	52.8	73.8	43.9	58.3	55.6	34.7	47.9	36.4	79.0	43.8
Monounsaturated fatty acids (MUFAs) [%]	29.0	11.7	46.4	24.8	32.9	31.7	37.1	43.6	10.7	18.8
Polyunsaturated fatty acids (PUFAs) [%]	18.2	14.5	9.7	16.9	11.5	33.7	15.0	20.0	10.3	37.4
(SFA+MUFA) (PUFA)^−1^	4.5	5.9	9.3	4.9	7.7	2.0	5.7	4.0	8.7	1.7
16:0 [%]	43.6	58.4	38.3	48.1	36.2	16.9	41.0	31.9	53.3	25.0
16:1n7	23.5	1.3	40.3	13.0	24.0	8.7	33.3	36.5	8.8	11.5

Total fatty acid content was normalized to cell number and is shown before dark incubation (T0) and after 3 months of dark incubation (T3) in five Antarctic benthic diatom species. Standard deviation was calculated according to Gaussian error propagation (n = 3). The percentage decrease in the total fatty acid content during this time was calculated. Saturated fatty acids (SFAs), monounsaturated (MUFA), and polyunsaturated (PUFA) fatty acids at T0 and T3 are given as the percentage of the total lipid content, as well as the ratio (SFA+MUFA)/(PUFA). In addition, the most abundant fatty acids detected in all strains, 16:0 and 16:1n7 are shown in the percentage proportion of all measured fatty acids.

## 4. Discussion

Since Antarctic benthic diatoms are regularly confronted with the polar night, we isolated five strains at Potter Cove, established clonal cultures, and investigated their physiological, biochemical, and cell biological traits after 3 months of darkness to better understand the underlying tolerance mechanisms.

### 4.1. Importance of taxonomy as baseline for ecophysiological investigations

However, the first step, prior to ecophysiological experiments, is always to carefully address the taxonomic position of freshly collected field material. Two of the investigated diatom species were morphologically and molecular-genetically identified as species only known from maritime Antarctica (*Chamaepinnularia gerlachei*) or the Southern Indian Ocean (*Navicula criophiliforma*). Two additional cultures belong to the newly described freshwater species *Planothidium wetzelii*, which is so far unknown in any other region of the world. Finally, one centric species was investigated in this study, which does not fit morphologically and genetically to any described *Melosira* species, and most probably represents a new taxon, which was not further investigated in the present study.

Due to a thorough identification of the investigated cultures, a restricted geographical distribution in the southern hemisphere could be verified for all of them, and some might even be endemic to the Antarctic region. Morphological variability of the newly described species *Planothidium wetzelii* shows that valves from environmental samples need to be complemented with molecular and morphological information gained by cultures. This case highlights once again the importance of integrative taxonomy for the investigation of diatom biodiversity. In addition to an integrated taxonomy, the establishment of clonal cultures is the prerequisite for a combined taxonomical and physiological investigation of benthic diatoms. The two *P. wetzelii* strains D300_015 and D300_025 exhibit some variability in valve morphology. They might be considered two separate species when just examined by light microscopy, based on their differences in shape and size. However, diatoms can exhibit phenotypic plasticity due to environmental changes (Andrejić et al., 2018) or when observed over long time in culture (Mohamad et al., 2022). Both strains represent the same species based on molecular markers and micromorphology, and the results from this study show very similar response patterns during dark incubation.

As a result of the inaccessibility of Antarctica, the diversity of benthic diatoms is still poorly known. Considerable taxonomic work needs to be done in polar regions, which can then be used as a baseline for physiological, biochemical, and cell biological studies, allowing important conclusions, such as, for example, if geographic boundaries and environmental conditions led to many endemic taxa.

### 4.2. Variable light conditions and photosynthesis

Phototrophic organisms in the polar regions such as benthic diatoms cope with strong diurnally and seasonally fluctuating light conditions. The review of Pavlov et al. (2019) comprehensively summarized the underwater light climate and all main controlling factors in the Arctic Kongsfjorden, one of the best studied polar field sites, which is discussed here as a representative high latitude coastal habitat since adequate data for Antarctica are missing. In addition to the seasonal changes from midnight sun to polar night, clouds, sea ice conditions, and the optical seawater properties further strongly influence dose and spectral composition of solar radiation penetrating into the water column, thus defining the underwater light conditions. In addition, input of local run-off and glacial meltwater introducing inorganic and organic matter into the system, and phytoplankton blooms at diverse times and locations. Together, these factors result in a complex underwater light climate with high variability in time and space (Pavlov et al., 2019). Although not comprehensively investigated, similar processes and physico-chemical properties can be assumed for Potter Cove (Hoffmann et al., 2019) and other bays around the Antarctic Peninsula. Polar benthic diatoms are additionally affected by huge amounts of particles released into the water column due to coastal glacier retreat and melt under global change conditions, resulting in increased turbidity and hence less available light (Hoffmann et al., 2019).

Photosynthesis is the essential mechanism for the energy metabolism and, thus, not only responsible for viability and survival of benthic diatoms, but also primarily dependent on light availability. All benthic diatom species examined in this study showed Y(II)_max_ of 0.57–0.63 before dark incubation as physiological marker for photosynthetic activity. Other studies on Antarctic benthic diatoms reported similar maximum Y(II)_max_ values between 0.6 and 0.7, indicating that the strains were in good physiological conditions prior to dark incubation (Longhi et al., 2003; Wulff et al., 2008a).

Prelle et al. (2022) used for their P–I curves various Antarctic benthic diatoms, including the species also investigated in the present study: *Navicula criophiliforma, Chamaepinnularia gerlachei*, and *P. wetzelii* strain D300_015, and demonstrated generally low light requirements, as light-saturated photosynthesis was reached at <70 μmol photons m^−2^ s^−1^. A similarly low light demand was observed for *Melosira* sp. and *P. wetzelii* strain D300_025 (data not shown). Other polar benthic diatoms are also known for their shade acclimation with low light compensation (I_c_) and light saturation points (I_k_) as well as steep initial slopes (α value) in their P–I curves (Wulff et al., 2008a). At the same time, they are also described as phototrophic microorganisms with a pronounced photophysiological plasticity, allowing them to cope with high solar radiation (Prelle et al., 2022).

Remarkable are the relatively high respiration rates, which were about as high as the net photosynthetic rates in some of the studied benthic diatom species, resulting in low ratios of NPP_max_: respiration, and which can be explained by co-occurring bacteria in the cultures. Overall, we cannot rule out that diatom-associated bacteria could affect the results of the P–I curves through their respiration, particularly when present in high cell numbers. However, bacteria and diatoms are known to have complex biotic interactions that affect each other's viability, and diatoms can even control their own phycosphere (microbiome; Amin et al., 2012). Therefore, the often-applied antibiotic treatment prior to experiments could potentially not only reduce essential bacteria but also affect diatom viability with unforeseen consequences for dark survival.

### 4.3. Physiological traits during darkness

Three months of darkness did not negatively affect Y(II)_max_ values in the studied benthic diatom species, which provides at least a crude measure for still-existing photosynthetic viability, which is confirmed by the oxygen data. Previous studies on the dark survival of polar diatoms showed a decrease in various photosynthetic parameters during dark incubation (Wulff et al., 2008a; Reeves et al., 2011; Karsten et al., 2012; Lacour et al., 2019). The only study on Antarctic benthic diatoms was performed by Wulff et al. (2008b) on semi-natural cultures with a dark period of 64 days. Their results revealed significant decreases in the parameters F_v_/F_m_, rETR (relative electron transport rate), and α (light-using efficiency).

All species revealed oxygen production after the dark period but to different degrees. While *C. gerlachei* and *Melosira* sp. showed a moderate decline in NPP_max_ (39.9 and 44.6%, respectively), the remaining species (*N. criophiliforma*, both *P. wetzelii* isolates) exhibited unaffected NPP_max_ values. Furthermore, the chlorophyll *a* data pointed to species-specific responses. While chlorophyll *a* decreased in *N. criophiliforma, C. gerlachei*, and *Melosira sp*. after 3 months of dark incubation, it remained more or less stable in both *P. wetzelii* strains. This observation is further supported by the TEM observations, which showed that some of the thylakoid membranes remained intact in this species. In contrast, in *N. criophiliforma*, much more degradation of the thylakoid system was observed with an increased number of plastoglobules. The lower NPP_max_ values can be related to the decline in chlorophyll *a* content as discussed by Wulff et al. (2008a) and Reeves et al. (2011), who explained the decrease in photosynthetic potential in Antarctic diatoms with a progressive degradation of the antenna complex and the reaction center. The decline in chlorophyll *a* concentration in some of the benthic diatom species can also be explained by chloroplast degradation after 3 months of darkness, but remarkably, this did not seem to have an effect neither on the physiological performance nor on the viability. Apparently, parts of the thylakoid membranes are degraded in darkness, but a functional part seems to remain intact and thus contributes to a basal level of photosynthetic activity. Kennedy et al. (2019) found similar results in Antarctic sea ice diatoms during 4 months of dark incubation and reported a rapid reduction of light-harvesting complexes and photosystems while maintaining photosynthetic capacity.

Applying SYTOX Green to the Arctic benthic diatoms *Surirella* cf. *minuta* and *Navicula directa* during 5 months of darkness indicated that an increasing number of cells exhibited damaged membranes over time in the first species (Karsten et al., 2019a), while the latter one was almost unaffected (Karsten et al., 2019b). In the present study, SYTOX Green staining was applied for the first time to Antarctic benthic diatoms, and the results are comparable to those on both Arctic species. While the percentage of damaged cells increased significantly in *N. criophiliforma, C. gerlachei, Melosira* sp., and *P. wetzelii* (D300_025) after 3 months of dark incubation, *P. wetzelii* (D300_015) maintained membrane integrity over this period of darkness. Although some diatom cells could not cope with longer darkness, others maintained viability and hence guaranteed the survival of the population after re-irradiation.

### 4.4. Cell biological traits during darkness

These are the first transmission electron micrographs of Antarctic benthic diatoms after dark treatment. They showed degraded chloroplasts in all benthic diatom species after 3 months of dark incubation with a markedly increased amount of plastoglobules (PGs) in the stroma of the plastids. PG appearance is a common response of plant cells to high light stress (Meier and Lichtenthaler, 1981) or any other stress, leading to a reduction of thylakoid membranes, as PGs contain the building blocks for thylakoids, including the enzymatic setting. The increase in PG in the Antarctic benthic diatoms can thus also be interpreted as a stress response; in this case, however, it is a response to long-term darkness. Schwarz et al. (2017) reported in the green microalga *Micrasterias* sp. how lipid droplets are released from the chloroplast into the cytoplasm during starvation, where they are degraded by autophagy. In addition, multivesicular bodies (MVBs) were also found in the cytoplasm after 7 weeks of starvation in darkness in *Micrasterias* sp., confirming autophagy (Schwarz et al., 2017). In the literature, it has been discussed that apoptosis and autophagy are both distinct processes but strongly interlinked (Mariño et al., 2014). In our study, the observable TEM structures are MVBs, which were present in *N. criophiliforma* and *P. wetzelii* (D300_015). Along with the numerous PGs and the degradation of the chloroplasts, this could indicate a mobilization of energy reserves by the autophagy of chloroplast components to survive the polar night. Furthermore, the chlorophyll *a* content per cell after 3 months of darkness strongly decreased in *N. criophiliforma, C. gerlachei*, and *Melosira sp*., supporting the ultrastructural results.

The cell biological data on Antarctic benthic diatoms confirm the few results on their Arctic pendants, which also reported, for example, a 30–50% decrease in chloroplast length (Karsten et al., 2012, 2019b). In addition, Wulff et al. (2008a) demonstrated condensed chloroplasts after dark treatment, which recovered within hours after re-exposure to light. Thus, no long-term damage appears to have been caused by the partial decomposition of chloroplasts during the prolonged darkness. According to Karsten et al. (2019b), the degradation of chloroplast compounds seems to be a key mechanism in Arctic benthic diatoms to survive the polar night and generate energy for their maintenance metabolism. The results of this study confirm that this is also a survival strategy for the examined Antarctic benthic diatoms.

### 4.5. Lipid content after 3 months of dark incubation

Both the light microscopic and the TEM images indicated significant differences in the lipid content of all species between control and after 3 months of dark incubation, which was additionally supported by the GC-MS analysis. *N. criophiliforma* and *Melosira* sp. consumed most of their lipid reserves in darkness, *C. gerlachei* depleted ~85% of their lipids, and both *P. wetzelii* strains still exhibited lipid droplets even after 3 months of darkness, which could suggest that this species might survive additional months in darkness. Enhanced lipid content, as well as the consumption of storage substances during darkness to maintain the cellular metabolism, have been described before for Arctic diatoms (Zhang et al., 1998; McMinn and Martin, 2013; Karsten et al., 2019b). Only a few studies on Arctic benthic diatoms quantified the decrease of lipid storage compounds during darkness (Schaub et al., 2017; Karsten et al., 2019a). Investigations on Antarctic benthic diatoms in this regard are entirely lacking so far.

Diatoms carry two pathways of β-oxidation for the breakdown of lipid compounds, the plant-like and the animal-like metabolic capabilities (Armbrust et al., 2004; Schaub et al., 2017). In the darkness, the mechanism of animal-like β-oxidation, located in the mitochondria, is upregulated and thus can provide energy from lipid storages during the polar night (Chauton et al., 2013), which probably allows diatoms to survive long periods of darkness, thereby maintaining their metabolism and the fundamental functions of their organelles (Armbrust et al., 2004). Thus, the results of the present study confirm the described metabolic pathway in Armbrust et al. (2004) and Chauton et al. (2013). In addition, in the more recent review of Leyland et al. (2020), the authors describe the storage of lipids in droplets and characterize these structures as organelles composed of a core of neutral lipids, mostly triacylglycerol (TAG), surrounded by a polar lipid monolayer. Most interestingly, lipid droplets can store not only reserves of energy but also membrane components, carbon skeletons, carotenoids, and even proteins (Leyland et al., 2020, and references therein).

While the membrane lipids of polar diatoms are mainly composed of polyunsaturated fatty acids in order to maintain membrane fluidity at low temperatures (Murata and Los, 1997), the storage lipids are mainly formed by triacylglycerol (TAG), which has three fatty acids that are mainly saturated or monounsaturated (Schaub et al., 2017). The latter authors carefully investigated qualitatively and quantitatively the lipid classes of the Arctic benthic diatom *Navicula perminuta* during 2 months of dark incubation. Schaub et al. (2017) demonstrated by the ratio of SFA and MUFA to PUFA that, indeed, lipid reserves were depleted, while membrane lipids remained unchanged, including thylakoid membranes. In the Antarctic *C. gerlachei, Melosira* sp., and both *P. wetzelii* strains, a decline in the ratio of fatty acids groups was observed, indicating enhanced degradation of storage lipids. In contrast, *N. criophiliforma* showed a slight increase in the ratio of fatty acids after 12 weeks, which points to a stronger increase in membrane degradation compared to lipid consumption. Apparently, both the consumption of storage lipids and the partial degradation of chloroplasts took place in the investigated Antarctic benthic diatoms during prolonged darkness in order to obtain energy for the maintenance metabolism.

## 5. Conclusion

In conclusion, all Antarctic benthic diatom species exhibited similar response patterns after 3 months of darkness. The combination of ecophysiological, biochemical, and cell biological data led to a better understanding of the underlying mechanisms. All benthic diatoms degrade parts of their chloroplasts and utilize their lipid energy reserves but at the same time maintained a functional photosynthetic apparatus that guarantees rapid recovery after re-irradiation. The combination of both mechanisms, presumably using storage lipids and degrading chloroplasts, is a key strategy for dark survival and hence for coping with the polar night.

## Data availability statement

The datasets presented in this study can be found in online repositories. The names of the repository/repositories and accession number(s) can be found in the article/Supplementary material.

## Author contributions

UK and JZ developed the concept for this manuscript. JZ sampled and OS isolated, purified, and established clonal cultures. DJ conducted the ecophysiological and biochemical parts of the manuscript. KS provided the light microscopic and scanning electron microscopic analyses. CP and AH undertook the transmission electron microscopy, combined the transmission electron micrographs, and edited the manuscript. KS and NA performed the taxonomic treatment. MG provided the GC-MS data. DJ and KS wrote the first version of the manuscript and prepared most of the figures. UK edited the first draft. All authors interpreted the data and edited and approved the final version of this manuscript.
