# Supplementary material for: Lipid degradation and photosynthetic traits after prolonged darkness in four Antarctic benthic diatoms, including the newly described species Planothidium wetzelii sp. nov
Source: Front Microbiol. 2023 Aug 31;14:1241826. doi: 10.3389/fmicb.2023.1241826 (PMC10500929; doi:10.3389/fmicb.2023.1241826)
Supplement: Supplementary file 1 [file Data_Sheet_1.PDF]

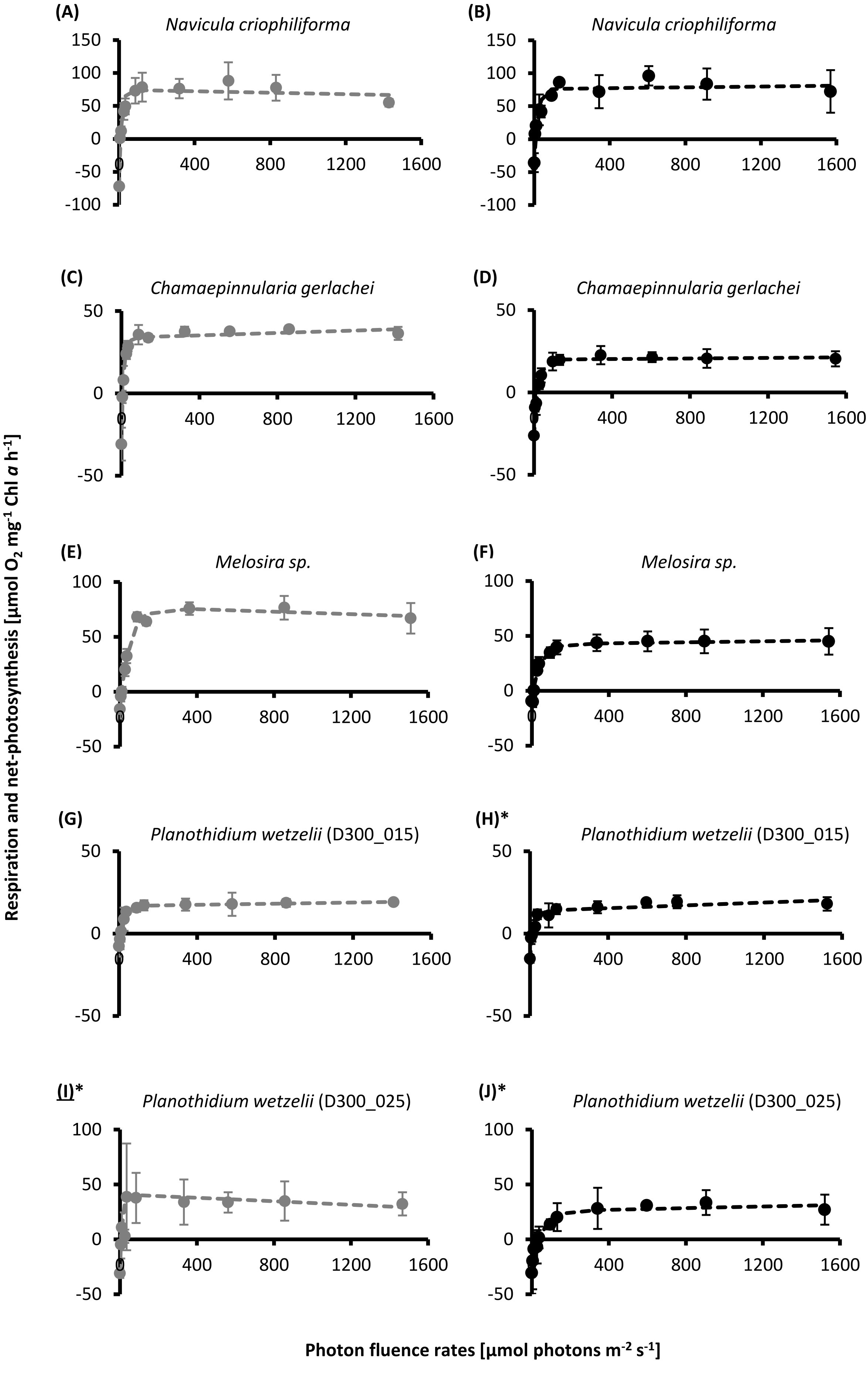

(A)
Navicula criophiliforma
(B)
Navicula criophiliforma
(C)
Chamaepinnularia gerlachei
(D)
Chamaepinnularia gerlachei
(E)
Melosira sp.
(F)
Melosira sp.
(G)
Planothidium wetzelii (D300_015)
(H)*
Planothidium wetzelii (D300_015)
(I)*
Planothidium wetzelii (D300_025)
(J)*
Planothidium wetzelii (D300_025)
150
100
50
0
-50
-100
0
400
800
1200
1600
Respiration and net-photosynthesis [µmol O2 mg-1 Chl a h-1]
Photon fluence rates [µmol photons m-2 s-1]

Supplementary Figure 1:

Photosynthesis-irradiance curves (PI-curves) of five benthic diatom strains form Antarctica. Each row shows a different strain, left column gives the control data of each culture (gray, T0), the right column the measurements after 3 months of dark incubation (T3) (black). **(A), (B)** *Navicula criophiliforma*. **(C), (D)** *Chamaepinnularia gerlachei*. ***(*E), (F)** *Melosira sp.*. **(G), (H)** *Planothidium wetzelii* (D300_015). **(I), (J)** *Planothidium wetzelii* (D300_025). The data points represent mean values ± SD (*n* = 4, if n = 3 marked *) and were fitted using the model of Walsby (1997).
