# Supplementary material for: Lipid degradation and photosynthetic traits after prolonged darkness in four Antarctic benthic diatoms, including the newly described species Planothidium wetzelii sp. nov
Source: Front Microbiol. 2023 Aug 31;14:1241826. doi: 10.3389/fmicb.2023.1241826 (PMC10500929; doi:10.3389/fmicb.2023.1241826)
Supplement: Supplementary file 2 [file Data_Sheet_2.PDF]

Supplementary Material

Lipid Degradation and Photosynthetic Traits after prolonged Darkness in four Antarctic Benthic Diatoms including the newly described species *Planothidium wetzelii* sp. nov.

Desirée P. Juchem[1]†, Katherina Schimani[2]†, Andreas Holzinger[3], Charlotte Permann[3], Nélida Abarca[2], Oliver Skibbe[2], Jonas Zimmermann[2], Martin Graeve[4], Ulf Karsten[1*]

[1]Applied Ecology and Phycology, Institute of Biological Sciences, Albert-Einstein-Strasse 3, University of Rostock, 18057 Rostock, Germany

[2]Botanischer Garten und Botanisches Museum Berlin, Freie Universität Berlin, Königin–Luise–Straße 6–8, 14195 Berlin, Germany

[3]Department of Botany, Functional Plant Biology, Sternwartestrasse 15, University of Innsbruck, Innsbruck 6020, Austria

[4] Alfred-Wegener-Institute Helmholtz-Center for Polar and Marine Research, Ecological Chemistry, Am Handelshafen 12, 27570 Bremerhaven, Germany

†These authors contributed equally to this work and share first authorship.

*** Correspondence:**
Corresponding Author
ulf.karsten@uni-rostock.de

Supplementary Table 1. Estimates of Evolutionary Divergence between *Planothidium* species (p-Distances) for the 18S V4 sequence.

	1	2	3	4	5	6	7	8	9	10	11	12	13	14	15	16	17	18	19	20	21	22	23	24	25
1. *P. wetzelii* D300_015																									
2. *P. wetzelii* D300_025	0.0																								
3. *P. wetzelii* D300_020	0.0	0.0																							
4. *P. wetzelii* D300_019	0.0	0.0	0.0																						
5. *P. tujii*	1.1	0.9	1.1	1.1																					
6. *P. victorii* D101_022	0.7	0.5	0.7	0.7	0.9																				
7. *P. victorii* B141	1.1	0.9	1.1	1.1	0.0	0.9																			
8. *P. victorii* B144	1.1	0.9	1.1	1.1	0.0	0.9	0.0																		
9. *P. victorii* D06_113	0.7	0.5	0.7	0.7	0.9	0.0	0.9	0.9																	
10. *P. victorii* D109_018	0.7	0.5	0.7	0.7	0.9	0.0	0.9	0.9	0.0																
11. *P. victorii* D06_014	0.9	0.7	0.9	0.9	0.2	0.7	0.2	0.2	0.7	0.7															
12. *P. victorii* type LCR-S-18-1-1	1.1	0.8	1.1	1.1	0.3	0.8	0.3	0.3	0.8	0.8	0.0														
13. *P. straubianum (= P.victorii)* B086_3	0.9	0.7	0.9	0.9	0.2	0.7	0.2	0.2	0.7	0.7	0.0	0.0													
14. *P. naradoense* D23_024	2.5	2.5	2.5	2.5	2.3	2.8	2.3	2.3	2.8	2.8	2.1	2.5	2.1												
15. *P. frequentissimum* D06_139	2.5	2.5	2.5	2.5	2.3	2.8	2.3	2.3	2.8	2.8	2.1	2.5	2.1	0.9											
16. *P. frequentissimum* D06_138	2.5	2.5	2.5	2.5	2.3	2.8	2.3	2.3	2.8	2.8	2.1	2.5	2.1	0.9	0.0										
17. *P. frequentissimum* TCC615	2.5	2.5	2.5	2.5	2.3	2.8	2.3	2.3	2.8	2.8	2.1	2.5	2.1	0.9	0.0	0.0									
18. *P. frequentissimum* LCR-S-2-1-1	3.2	3.2	3.2	3.2	2.9	3.5	2.9	2.9	3.5	3.5	2.6	2.9	2.6	1.2	0.0	0.0	0.0								
19. *P. lanceolatum* D06_047	4.6	4.6	4.6	4.6	4.8	5.3	4.8	4.8	5.3	5.3	4.8	5.9	4.8	4.1	3.9	3.9	3.9	4.9							
20. *P. lanceolatum* B146	5.3	5.3	5.3	5.3	5.5	5.9	5.5	5.5	5.9	5.9	5.5	6.2	5.5	4.8	4.6	4.6	4.6	5.2	0.7						
21. *P.* cf. *subantarcticum* D17_002	7.8	7.6	7.8	7.8	7.6	8.0	7.6	7.6	8.0	8.0	7.6	9.3	7.6	7.4	7.1	7.1	7.1	7.0	6.2	6.7					
22. *P. taeansa* D26_002	4.8	4.6	4.8	4.8	4.6	5.0	4.6	4.6	5.0	5.0	4.6	5.6	4.6	4.4	3.7	3.4	3.4	4.3	2.7	3.4	6.9				
23. *P. cryptolanceolatum* D26_017	4.3	4.1	4.3	4.3	4.3	4.6	4.3	4.3	4.6	4.6	4.3	5.3	4.3	4.6	4.1	4.1	4.1	5.2	2.7	3.4	6.2	2.7			
24. *P. cryptolanceolatum* D31_010	4.3	4.1	4.3	4.3	4.3	4.6	4.3	4.3	4.6	4.6	4.3	5.3	4.3	4.6	4.1	4.1	4.1	5.2	2.7	3.4	6.2	2.7	0.0		
25. *P. cryptolanceolatum* Ko8A0610-1	4.3	4.1	4.3	4.3	4.3	4.6	4.3	4.3	4.6	4.6	4.3	5.3	4.3	4.6	4.1	4.1	4.1	5.2	2.7	3.4	6.2	2.7	0.0	0.0	
26. *P. suncheonmanense* Ko0408	5.0	5.0	5.0	5.0	5.0	4.8	5.0	5.0	4.8	4.8	5.0	6.2	5.0	5.0	4.8	4.8	4.8	6.1	5.3	5.7	8.3	5.7	5.0	5.0	5.0

Supplementary Table 2. Estimates of Evolutionary Divergence between *Planothidium* species (p-Distances) for the *rbc*L sequence

	1	2	3	4	5	6	7	8	9	10	11	12	13	14	15	16	17	18	19	20	21	22	23	24	25
1. *P. wetzelii* D300_015																									
2. *P. wetzelii* D300_025	0.0																								
3. *P. wetzelii* D300_020	0.0	0.0																							
4. *P. wetzelii* D300_019	0.0	0.0	0.0																						
5. *P. tujii*	0.5	0.5	0.5	0.5																					
6. *P. victorii* D101_022	0.6	0.6	0.6	0.6	0.5																				
7. *P. victorii* B141	0.5	0.5	0.5	0.5	0.4	0.5																			
8. *P. victorii* B144	0.5	0.5	0.5	0.5	0.4	0.5	0.2																		
9. *P. victorii* D06_113	0.6	0.6	0.6	0.6	0.5	0.0	0.5	0.5																	
10. *P. victorii* D109_018	0.7	0.7	0.7	0.7	0.6	0.0	0.6	0.6	0.0																
11. *P. victorii* D06_014	0.6	0.6	0.6	0.6	0.5	0.0	0.5	0.5	0.0	0.0															
12. *P. victorii* type LCR-S-18-1-1	0.8	0.8	0.8	0.8	0.8	0.3	0.5	0.8	0.3	0.3	0.3														
13. *P. straubianum (= P.victorii)* B086_3	0.5	0.5	0.5	0.5	0.6	0.7	0.6	0.6	0.7	0.8	0.7	1.0													
14. *P. naradoense* D23_024	2.2	2.2	2.2	2.2	2.0	2.1	2.2	2.2	2.1	2.2	2.1	2.0	1.7												
15. *P. frequentissimum* D06_139	1.8	1.8	1.8	1.8	1.8	1.7	1.8	1.8	1.7	1.8	1.7	2.8	1.9	2.0											
16. *P. frequentissimum* D06_138	1.8	1.8	1.8	1.8	1.8	1.7	1.8	1.8	1.7	1.8	1.7	2.8	1.9	2.0	0.0										
17. *P. frequentissimum* TCC615	2.1	2.1	2.1	2.1	2.1	2.0	2.1	2.1	2.0	2.1	2.0	3.5	2.2	2.4	0.1	0.1									
18. *P. frequentissimum* LCR-S-2-1-1	2.3	2.3	2.3	2.3	2.6	2.1	2.3	2.6	2.1	2.1	2.1	2.3	2.6	2.1	0.5	0.5	0.4								
19. *P. lanceolatum* D06_047	3.8	3.8	3.8	3.8	3.7	3.9	3.8	3.8	3.9	4.0	3.9	4.6	3.6	3.7	3.9	3.9	4.1	4.7							
20. *P. lanceolatum* B146	3.7	3.7	3.7	3.7	3.6	3.9	3.7	3.7	3.9	3.9	3.9	4.6	3.5	3.5	3.8	3.9	4.1	4.7	0.1						
21. *P.* cf. *subantarcticum* D17_002	3.6	3.6	3.6	3.6	3.6	3.9	3.7	3.7	3.9	3.9	3.9	4.1	3.3	3.7	3.7	3.8	4.2	3.9	1.9	2.0					
22. *P. taeansa* D26_002	3.3	3.3	3.3	3.3	3.3	3.3	3.4	3.4	3.4	3.4	3.4	4.1	3.2	3.6	3.4	3.5	3.9	3.9	2.5	2.4	1.7				
23. *P. cryptolanceolatum* D26_017	3.0	3.0	3.0	3.0	3.0	3.0	3.1	3.1	3.1	3.1	3.1	4.1	2.9	3.5	3.3	3.4	3.9	3.9	2.6	2.5	1.8	0.5			
24. *P. cryptolanceolatum* D31_010	3.0	3.0	3.0	3.0	3.0	3.0	3.1	3.1	3.1	3.1	3.1	4.1	2.9	3.5	3.3	3.4	3.9	3.9	2.6	2.5	1.8	0.5	0.0		
25. *P. cryptolanceolatum* Ko8A0610-1	3.0	3.0	3.0	3.0	3.0	3.0	3.1	3.1	3.1	3.1	3.1	4.1	2.9	3.5	3.3	3.4	3.9	3.9	2.6	2.5	1.8	0.5	0.0	0.0	
26. *P. suncheonmanense* Ko0408	6.2	6.2	6.2	6.2	5.9	5.9	6.2	6.2	5.9	6.0	5.9	6.6	6.2	5.6	5.6	5.6	6.2	6.0	5.3	5.3	6.0	5.7	5.6	5.6	5.6
